# Conformational changes and catalytic inefficiency associated with Mot1-mediated TBP–DNA dissociation

**DOI:** 10.1093/nar/gky1322

**Published:** 2019-01-15

**Authors:** Gregor Heiss, Evelyn Ploetz, Lena Voith von Voithenberg, Ramya Viswanathan, Samson Glaser, Peter Schluesche, Sushi Madhira, Michael Meisterernst, David T Auble, Don C Lamb

**Affiliations:** 1 Department für Chemie, Center for Nanoscience (CeNS), Center for Integrated Protein Science Munich (CIPSM) and Nanosystems Initiative Munich (NIM), Ludwig-Maximilians-Universität, München 81377, Germany; 2 Department of Biochemistry and Molecular Genetics, University of Virginia Health System, Charlottesville, VA 22908, USA; 3 Institut für Molekulare Tumorbiologie, Westfälische Wilhelms-Universität, Münster 48149, Germany

## Abstract

The TATA-box Binding Protein (TBP) plays a central role in regulating gene expression and is the first step in the process of pre-initiation complex (PIC) formation on promoter DNA. The lifetime of TBP at the promoter site is controlled by several cofactors including the Modifier of transcription 1 (Mot1), an essential TBP-associated ATPase. Based on ensemble measurements, Mot1 can use adenosine triphosphate (ATP) hydrolysis to displace TBP from DNA and various models for how this activity is coupled to transcriptional regulation have been proposed. However, the underlying molecular mechanism of Mot1 action is not well understood. In this work, the interaction of Mot1 with the DNA/TBP complex was investigated by single-pair Förster resonance energy transfer (spFRET). Upon Mot1 binding to the DNA/TBP complex, a transition in the DNA/TBP conformation was observed. Hydrolysis of ATP by Mot1 led to a conformational change but was not sufficient to efficiently disrupt the complex. SpFRET measurements of dual-labeled DNA suggest that Mot1’s ATPase activity primes incorrectly oriented TBP for dissociation from DNA and additional Mot1 in solution is necessary for TBP unbinding. These findings provide a framework for understanding how the efficiency of Mot1’s catalytic activity is tuned to establish a dynamic pool of TBP without interfering with stable and functional TBP-containing complexes.

## INTRODUCTION

The Snf2/Swi2 ATPases comprise a large group of evolutionarily conserved enzymes that catalyze the remodeling of protein–DNA complexes involved in all of the fundamental processes of DNA metabolism (1–4). The RecA folds that define the catalytic core are related to helicase motor domains (5), and among a small number of enzymes that have been explicitly tested, adenosine triphosphate (ATP) hydrolysis has been shown to induce DNA translocation (6–9). Functional specificity is conferred through the coupling of DNA translocation to effector domains that mediate disruption of protein–DNA interactions, which can lead to structural reorganization, protein–DNA complex disassembly, or even the establishment of new protein–DNA complexes (10,11). Most Snf2/Swi2 ATPases function as components of multimeric protein complexes (12,13), and the biochemical and structural complexity has been explored in depth for only some of them.

Modifier of transcription 1 (Mot1) is a member of the Snf2/Swi2 family and it uses ATP hydrolysis to dissociate TATA-box binding protein (TBP)–DNA complexes *in vitro* (14). It is an essential gene in yeast (15–17) and is evolutionarily conserved in yeast as well as in higher eukaryotes. Mot1 contributes to the establishment of the dynamic behavior of TBP on a global scale (18,19) and, as a consequence, defects in Mot1 result in large-scale changes to transcription (20–23). Mot1 behaves as a transcriptional repressor at inactive stress-responsive genes, which tend to have high affinity TBP-binding sites in their promoters (24–26). Conversely, Mot1 functions directly as a transcriptional activator at many promoters that lack strong TATA sequences (20,21,24,27). A number of models for Mot1’s paradoxical activation activity have been proposed, including providing a pool of unbound TBP for use in transcription and disassembly of transcriptionally inactive TBP-containing complexes that do not contain appropriate levels of other essential general transcription factors (28).

Structurally, a monomer of Mot1 interacts with the convex surface of TBP via an N-terminal α-helical array of HEAT repeats (29). A latch domain extends from the N-terminal region and interacts with TBP’s DNA binding surface; the latch improves the efficiency of TBP–DNA dissociation *in vitro* and may allow Mot1 to function as a chaperone for TBP that is not associated with chromatin (29). When bound to the TBP–DNA complex, the Mot1 C-terminal ATPase domain is positioned on DNA to the ‘upstream’ side of TBP (with respect to the start site of transcription) such that ATP hydrolysis would allow the domain to translocate along DNA (29,30). ATP hydrolysis-driven DNA translocation is thus thought to underlie the TBP–DNA dissociation mechanism, although translocation by the Mot1 ATPase has not been directly observed and Mot1 has no detectable ability to translocate along DNA processively (30,31). The severely bent DNA conformation in the DNA/TBP complex may also be involved in the Mot1 catalytic mechanism (32). Although Mot1 can function as a single polypeptide, its *in vivo* function partially overlaps with the NC2 heterodimer (33). The NC2–TBP complex encircles DNA (34) and it can diffuse along a DNA template without dissociating from it (35). Complexes of Mot1, NC2, TBP and DNA have been identified and characterized as well (30,36,37). NC2 stabilizes Mot1 binding in the absence of ATP, but Mot1 can dissociate complexes both with and without NC2 (30,36). Thus, as far as is currently known, once Mot1 interacts with TBP–DNA, its dissociation activity is not notably modified or altered by other factors.

In contrast, the binding of Mot1 to TBP competes with other factors that interact with overlapping surfaces on TBP, including transcription factor IIA (TFIIA), TBP-associated factor 1 (Taf1) and TFIIB-related factor 1 (Brf1) (29,38,39). In addition, Mot1’s ability to dissociate TBP from DNA must be tuned kinetically to balance the biological requirement for generating a dynamic pool of TBP in the nucleus with the requirement for TBP as a central structural component of the transcription pre-initiation complex (PIC), which is very stable *in vitro* (40,41). An overly efficient or unregulated TBP–DNA dissociation activity would prevent PIC formation and diminish transcription globally (42). Indeed, Mot1 overexpression inhibits cell growth due to enhanced and unbalanced activity toward TBP (14,43). Consistent with this, recent evidence has suggested that Mot1 does not efficiently dissociate high affinity TBP–DNA complexes (44) and, in addition, Mot1 has been implicated in the turnover of nonspecific or transcriptionally inactive interactions with DNA (45).

Collectively, the results to date provide a rich picture of the biological roles of Mot1, its enzymatic capabilities and the structural basis for carrying them out. However, prior biochemical studies have relied on ensemble measurements and, as a result, there is relatively little information available regarding the dynamic behavior of individual complexes or the time-dependent fates of TBP-containing complexes that are resistant to Mot1-mediated dissociation. To better understand the functional roles of Mot1, we utilized single-pair Förster resonance energy transfer (spFRET) in combination with total internal reflection fluorescence microscopy (TIRFM). SpFRET can provide distance information between pairs of fluorophores and thus identify different conformational states and can also be used to investigate the dynamic behavior of biomolecules and complexes (46,47). Here, we report, using spFRET, evidence for conformational transitions in TBP–DNA complexes upon Mot1 binding, including the effects of nucleotide addition, which had not been previously reported. A Hidden Markov Model (HMM) approach revealed the pathways for conformational interconversion and the relative occupancies of each conformational state. We also show that two Mot1 molecules are required to dissociate TBP from DNA *in vitro*. These results provide a framework for understanding how a Snf2/Swi2 ATPase has been tuned catalytically to meet the competing demands of a targeted general transcription factor that must be dynamically available for transcription complex assembly and yet stably bound to DNA at appropriate times and locations to promote transcription.

## MATERIALS AND METHODS

### Recombinant proteins

Recombinant TBP^S61C,C78A,C164A^ from *Saccharomyces cerevisiae* was expressed in *Escherischa coli* BL21-Codon Plus DE3 RIL. The cells were lysed by sonication in lysis buffer (25 mM Tris, 500 mM NaCl, 50 mM ammonium acetate, 10% glycerol, pH 8) with 10 mM tris(2-carboxyethyl)phosphine (TCEP), 10 mM phenylmethylsulfonyl fluoride (PMSF). Purification was performed in a three-step process via affinity chromatography and gel filtration. In a first step, soluble proteins were bound to a Ni-affinity chromatography column, which was washed with lysis buffer including 5 mM imidazole. TBP was eluted by the addition of lysis buffer containing 250 mM imidazole. Further purification was performed on a heparin chromatography column with a gradient from 250 mM NaCl to 1 M NaCl in lysis buffer. TBP was eluted from the column at a concentration of 600–800 mM NaCl. In a third step, TBP was purified via size-exclusion chromatography on a superose 6 column in TBP buffer (5 mM 4-(2-hydroxyethyl)-1-piperazineethanesulfonic acid (HEPES), 40 mM ammonium sulfate, 10 μM ZnCl_2_, 20% glycerol, pH 7.3). TBP was site-specifically labeled with the thiol-reactive group of the maleimide-coupled fluorophores Atto532 and Atto647N as recommended (ATTO-TEC). Expression and purification of recombinant Mot1, NC2, and PC4 was performed as described previously (35,43,48).

### Oligonucleotides

Oligonucleotides used in this study were derived from the TATA-box sequence of H2B promoters and the adenovirus major-late promoter (for sequences see Supplementary Figure S1). Fluorescently labeled DNA strands were purchased (IBA Lifesciences, Metabion) and annealed in TE (10 mM Tris, pH 8, 1 mM ethylenediaminetetraacetic acid) by heating to 95°C and cooling to room temperature at 1°C per min.

### Surface preparation

SpFRET experiments on the surface were performed in quartz prism-based flow-chambers. For surface passivation, the prisms were treated with aminosilane and polyethylene glycol (PEG) as described previously (49). Attachment of PEG included 1–3% biotinylated PEG molecules. Flow chambers were built using specifically designed channels cut in Nescofilm and sandwiched between a thoroughly cleaned coverglass and the prism. For details, see Schluesche *et al.* (35).

### Experimental prism-type TIRF setup

All FRET experiments were carried out on a custom-built prism-type TIRF setup built using a TE2000-U (Nikon) base. The sample was excited by an Ar/Kr ion laser (488 nm, Coherent), a Nd/YAG laser (532 nm, CrystaLaser) and a HeNe laser (633 nm, Laser 2000). Laser intensity as well as excitation duration (15–300 ms) was controlled by an acousto-optical tunable filter (AOTF.nC-TN, Pegasus Optik), which was synchronized to an EM-CCD camera (iXon+ 897, Andor) used for dual-color detection through a custom-built, real-time control unit. Fluorescence was collected by a 60× water immersion objective (CFI Plan Apo VC, Nikon, N.A. 1.2), spectrally separated by a dichroic mirror (630 DCXR, AHF) and filtered by the bandpass filters HQ550/88 and HQ715/150 (AHF). Fluorescence was detected on the EM-CCD camera split into two channels according to wavelength and an image series was recorded (Supplementary Figure S2A).

### SpFRET TIRF experiments

#### Sample preparation

Measurements were performed in quartz flow chambers with passivated surfaces as described above, in which the 5′-end of the DNA was immobilized via a biotin-streptavidin-biotin linkage. The prisms were incubated with 0.3 mg/ml streptavidin in phosphate buffered saline (PBS) for 10 min and washed with PBS. DNA complexes were flown into the chamber in pM concentrations in working buffer (10 mM Tris, 60 mM KCl, 5 mM MgCl_2_, 10% glycerol, 0.25 mg/ml bovine serum albumin, 2.5 mM dithiothreitol, pH 8.2). DNA/TBP complex formation was observed by dilution of a pre-incubated mixture of 10 nM DNA 1-Atto647N and 15 nM TBP-Atto532 (15 min, RT) to ∼100 pM. To observe conformational changes within the DNA, a mixture of 5 nM DNA 3-Atto532-Atto647N and 25 μM TBP (or 750 nM TBP-Atto488, and 25 μM TFIIA) was pre-incubated at room temperature for 15 min. For DNA/TBP/Mot1 complex formation, 12.5 nM Mot1 was added after initial incubation and was allowed to incubate for at least another 15 min at RT. As 12.5 nM is well above the affinity of Mot1 for TBP DNA (∼1.5 nM (50)), the majority of complexes have a Mot1 molecule bound under these conditions. For dissociation experiments, 10 nM DNA 2-Atto532 was pre-incubated with 10 nM TBP-Atto647N (15 min., RT) to avoid artifacts due to FRET and thus donor photobleaching. For ternary complex formation in both experiments, the incubation period was extended by an additional 15 min with 12.5 nM Mot1. The incubated sample was diluted to a concentration of ∼100 pM and flushed into the flow chamber and incubated for ∼10 min. Unbound complexes were then removed by washing the chamber with additional working buffer. For dissociation measurements, a solution of 1 mM ATP and 3 nM Mot1 in working buffer (when not indicated otherwise) was flushed into the flow chamber during the recording.

#### Fluorescence measurements and detection

For three-color experiments with labeled Mot1, the laser excitation switched frame-by-frame between 488 and 532 nm. The fluorescence from Alexa488 and Atto532 were recorded in the green channel in alternate frames while the fluorescence of the Atto647N dye due to FRET was simultaneously recorded in the red channel. In dissociation experiments, the whole camera chip was used for the detection of TBP-Atto647N with an excitation time of 30 ms per frame. In this case, only the first 10 frames were imaged with 532 nm excitation to ensure complex formation and, during the following frames, Atto647N was excited directly by the HeNe laser. To obtain a high time resolution (15 ms) for dynamic analysis of dual-labeled DNA, only half of the camera chip was read and only 532 nm excitation was used (i.e. no alternating laser excitation for these measurements).

### SpFRET MFD experiments

For multi-parameter fluorescence detection (MFD) measurements, sample preparation was performed as described for spFRET TIRF experiments. The complexes were diluted to <50 pM to minimize the probability of having two or more molecules in the confocal observation volume at a time and measured for a period of 2 h. The experiments were performed on a homebuilt confocal microscope described in Mapa *et al.* (51). Pulsed interleaved excitation was achieved using picosecond pulsed interleaved lasers (PicoQuant, Berlin) with the wavelengths 532 and 640 nm. A laser power of 100 μW for each laser, measured at the back aperture of the objective, was used.

### Immobilized template assays

The experimental results from the ensemble immobilization measurements discussed in the Supplementary Results R1 were performed using reaction conditions, DNA, recombinant protein concentrations and western blotting for TBP exactly as described in (44).

### Data analysis

Data analysis was performed using home-written MATLAB software. The software for the MFD experiments is freely available online (52) and for TIRF is available upon request. For spFRET TIRF experiments, a transformation map for the separate channels on the camera chip was calculated based on images of fluorescent beads. The intensities of individual molecules in both channels could then be extracted and intensity-based FRET values were calculated. Correction factors for spectral crosstalk and relative detection efficiencies could be determined for individual molecules, when acceptor photobleaching occurred before donor photobleaching. For three-color experiments, the separation of the recorded fluorescence intensities was based on the excitation scheme. Complexes containing Mot1 were identified by the presence of fluorescence in the green channel after 488 nm excitation. Direct excitation of the Atto532 dye by 488 nm excitation was corrected for by subtracting a fraction of the Atto532 fluorescence after 532 nm excitation from the fluorescence recorded after illumination at 488 nm.

**Figure 1.**
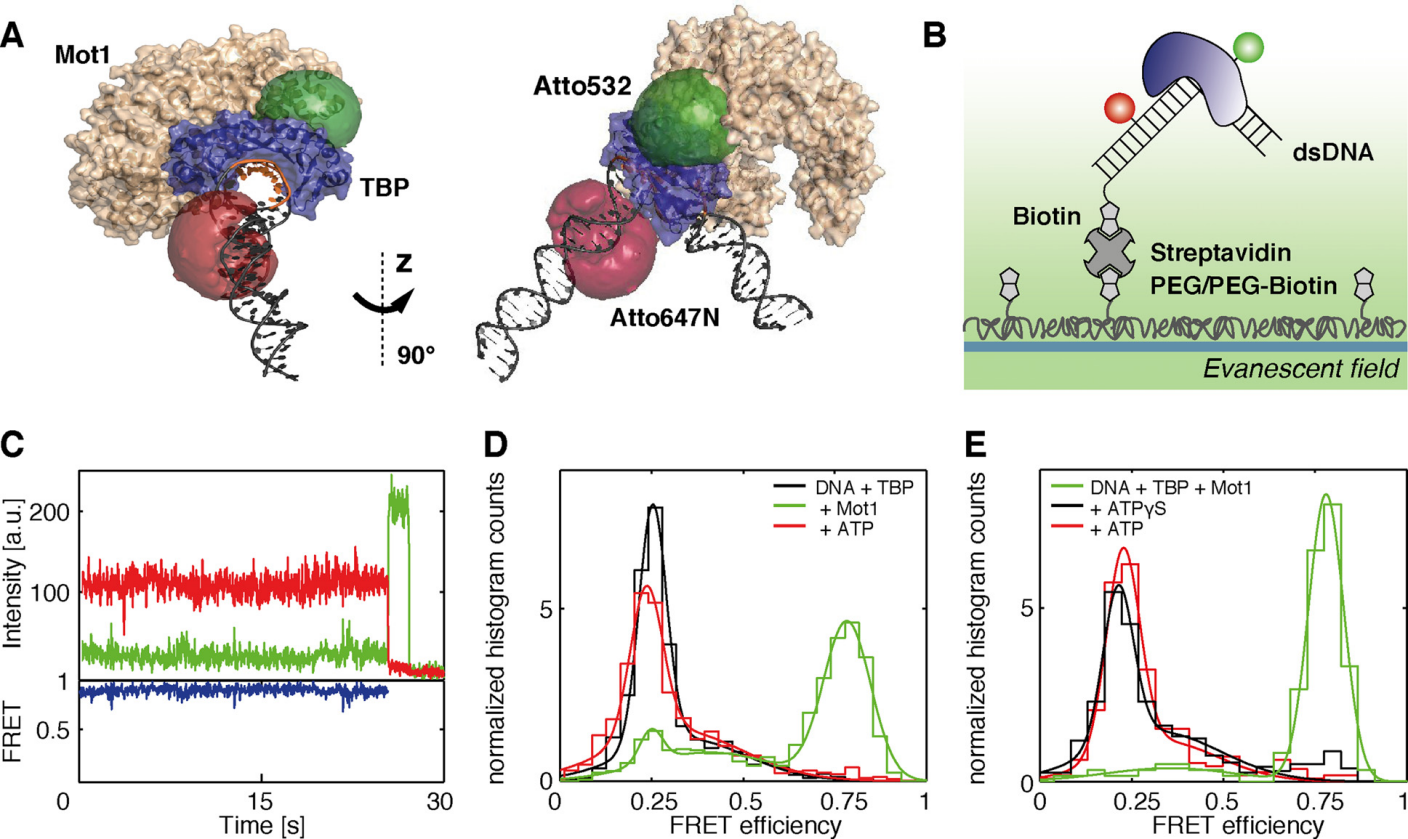
Conformation of the DNA/TBP complex in the absence and presence of Mot1 observed by intermolecular spFRET. (**A** and **B**) Structure and labeling positions within the investigated Mot1/DNA/TBP complex visualized by Pymol. TBP is depicted in dark blue, the Mot1 N-terminal domain (NTD) in beige. The TATA sequence is marked in orange. Fluorescent labels are located within their sterically accessible volumes shown as red and green half cycles. The model structure is based on PDB structures 5FMF and 4WZS. For technical details, see ‘Supplementary Materials and Methods’ section. (A) Structures of single-labeled double-stranded DNA (dsDNA) in complex with TBP and Mot1. The DNA is labeled upstream at position +13 with respect to the transcription start site. TBP is labeled at residue 61. In complexes with DNA2, the DNA is labeled with Atto532 and TBP with Atto647N. In complexes with DNA1, the dyes are switched. (B) Immobilization strategy for single-molecule FRET imaging on DNA/TBP by TIRF microscopy. (**C**) A single-molecule time trace of a static DNA/TBP/Mot1 complex. Upper panel: the donor and acceptor intensities are shown in green and red, respectively. The donor intensity increases after single step acceptor photobleaching. Lower panel: the calculated FRET efficiency is shown in blue. (**D**) Molecule-wise FRET efficiency histograms of TBP complexes on H2B promoter DNA 1. Black: pre-assembled DNA/TBP complexes. Green: pre-assembled DNA/TBP/Mot1 complexes. Red: ternary DNA/TBP/Mot1 complexes after addition of 1 mM ATP. The ATP addition was performed in the same flow-chamber under the same buffer conditions as the measurements of the DNA/TBP/Mot1 complex. For parameters of the Gaussian fit, see Table 1. (**E**) Molecule-wise FRET efficiency histogram of TBP/Mot1 complexes on H2B promoter DNA 1. The complexes were pre-incubated at concentrations of 10 nM DNA 1 and 10 nM TBP, 12 nM Mot1 and then diluted to final concentrations of ∼50 pM. Green: pre-assembled DNA/TBP/Mot1 complexes. Black: DNA/TBP/Mot1 complexes after addition of 1 mM ATPγS. Red: DNA/TBP/Mot1 complexes after addition of 1 mM ATP following the addition of ATPγS. The number of molecules in each experiment is given in Supplementary Table S1. For parameters of the Gaussian fit used to guide the eye, see Supplementary Table S3.

The single-pair FRET histograms (Figure 1 and Supplementary Figure S4) where fit to Gaussian distributions. The low FRET population of the TBP/DNA data could be described with a main Gaussian population ( E = 0.25, σ = 0.04) and a small shoulder (E = 0.18, σ = 0.07). For simplicity, we have combined the areas of the two Gaussians in Table 1. A second population had a FRET efficiency of 0.34 and width of 0.13. From the DNA/TBP/Mot1 data, we could determine the peak and width of the fourth Gaussian (peak of ∼ 0.78 and a width of 0.07). Using these values, we then performed a global fit that assumed four states with fixed peak positions and widths where only the amplitudes were allowed to vary. A small shift in peak FRET efficiencies (e.g. from 0.25 to 0.26 for the DNA/TBP population) due to slightly differing background correction factors was allowed when necessary, as indicated in Table 1 and Supplementary Table S3. R^2^ values of better than 0.98 were obtained for all fits. The fit parameters are given in Table 1 and Supplementary Table S3.

**Table 1.** Parameters of the normal distribution used to fit the molecule-wise FRET histogram shown in Figure 1D

	S_1_			S_2_			S_3_		
	A_1_	μ_1_	σ_1_	A_2_	μ_2_	σ_2_	A_3_	μ_3_	σ_3_
DNA/TBP	72	25	4	27	34	13	-	-	-
DNA/TBP/Mot1	6	24	4	28	34	13	65	78	7
DNA/TBP/Mot1+ATP	49	24	4	43	32	13	8	77	7

The FRET efficiency is measured between the downstream labeled TATA box and labeled TBP. The mean FRET efficiency, μ, and standard deviation, σ, are given as FRET efficiency (in %). The relative areas of the Gaussian distribution, A, is given in %.

TBP dissociation from DNA was measured by comparing the effective time a molecule exhibited fluorescence after ATP addition with the time a molecule showed fluorescence before photobleaching determined for each measurement individually. Both curves were fit using mono-exponential decays,

(1) \begin{equation*}f\ \left( {{x_i}} \right) = {e^{ - {k_i}t}}\ \end{equation*}

and the difference in the obtained decay rates (*k*_2_ *− k*_1_) was attributed to dissociation of TBP.

FRET efficiencies of dynamic dual-labeled DNA molecules in complex with additional cofactors were further analyzed using an HMM, which was implemented based on the HMM toolbox written by Kevin Murphy (1998). Molecules were manually classified as static or dynamic. For each molecule showing dynamic switching between several FRET states, an individual HHM model with three states was trained. We chose an HMM model with three states, as this model contained the lowest number of states describing the observed data based on the maximum likelihood and visual inspection of the data (‘Supplementary Material and Methods’ section, Supplementary Figure S3). Convergence was assumed when the change of likelihood between iterations was less than 1 × 10^−9^. Each transition was superimposed as a 2D-Gaussian with a fixed width into the TDP. From there, clusters of subpopulations were selected and a histogram of dwell times for the individual states was generated. Decay rates were obtained from mono-exponential fits to the data.

Statistics and imaging settings for the various experiments can be found in Supplementary Table S1 and Supplementary Table S2, respectively.

## RESULTS

### Conformational states of the DNA/TBP complex upon addition of Mot1

Mot1 is known to bind to TBP via an α-helical array and a latch like structure (29,48,53). To investigate the effect of Mot1 binding on the conformation of the DNA/TBP complex, spFRET was monitored between site-specifically labeled TBP and an H2B promoter construct (DNA1, Supplementary Figure S1) using a TIRF microscope (Figure 1A and Supplementary Figure S2A). It has been shown previously that fluorescently labeled TBP at position 61 is still functional (54,55). In contrast to ensemble measurements where unbound Mot1 or TBP are always present in solution, here individual immobilized complexes were observed and the amount of additional protein in solution could be controlled using a flow cell. The majority of ∼60% of the ternary DNA/TBP/Mot1 complexes displayed a static FRET signal. An exemplary intensity and FRET efficiency time trace of a static DNA/TBP/Mot1 complex is shown in Figure 1C and Supplementary Figure S2B. Supplementary Figure S2C shows two exemplary dynamic traces. Interestingly, the fraction of static complexes increased to over 90% and the absolute number of complexes was not significantly altered after ATP addition to ternary DNA/TBP/Mot1 complexes.

The molecule-wise FRET efficiency histogram of static DNA/TBP complexes in the absence of Mot1 can be described by two Gaussian distributions. The majority population (72%) is found in a low FRET state (0.25 FRET efficiency) while 27% of the complexes displayed an intermediate FRET efficiency of 0.34 (Table 1). For spFRET measurements using the AdML promoter, we found the low FRET state corresponded to a conformation with fully bound TBP, while the intermediate FRET population was due to TBP bound with the incorrect orientation on the TATA box (56). For the H2B promoter, we observed changes in this region of the spFRET histogram, which may suggest an influence of TFIIA on the redistribution of TBP orientation or positioning of TBP on the H2B TATA box (Supplementary Figure S4 and Supplementary Table S3). Interestingly, the addition of NC2 to the DNA/TBP/Mot1 or the DNA/TBP/Mot1/TFIIA complex does not alter the FRET efficiency distribution (Supplementary Figure S4) nor stability of the complex. Figure 1D compares the FRET efficiency histograms of DNA/TBP complexes to DNA/TBP/Mot1 ternary complexes. After incubation of the DNA/TBP complexes with Mot1, the number of complexes showing a FRET efficiency of 0.25 decreased and 65% of DNA/TBP/Mot1 complexes adopted a conformation with a FRET efficiency of 0.78. Although the binding of Mot1 was not directly monitored, the conformational change of the complex indicates formation of the ternary DNA/TBP/Mot1 complex. No crystal structure of the full Mot1-TBP-DNA complex is currently available, but Butryn *et al*, 2015 (30) reported the structure of the Mot1 N-terminal domain-TBP-NC2–DNA complex. The DNA in the quaternary complex was modestly unbent compared to TBP–DNA alone. A change in the DNA conformation could be consistent with the proposal that Mot1 mediates dissociation of TBP–DNA via a release of spring-like tension in the DNA (32).

Upon addition of 1 mM of ATP to the ternary DNA/TBP/Mot1 complex, the high FRET state disappeared and the majority of the complexes (50%) were found in the low FRET state (0.25 FRET efficiency). This conformational rearrangement indicates functional ATPase activity of Mot1 in the ternary complexes. The relative number of molecules with intermediate FRET efficiency was not strongly altered when Mot1 or ATP were added (27, 28 and 43% for DNA/TBP, DNA/TBP/Mot1 and DNA/TBP/Mot1 + ATP, respectively) (Table 1).

The spFRET histograms of the DNA/TBP and the DNA/TBP/Mot1 complex after addition of ATP look very similar, which could be explained by dissociation of Mot1 upon addition of ATP. To test this hypothesis, we performed three-color experiments with fluorescently labeled Mot1. Mot1 was labeled with Alexa488 and its presence was determined via the measured fluorescence intensity (Supplementary Figure S5). Complexes containing labeled Mot1 showed two subpopulations in the FRET distribution, as observed in the two-color FRET experiments (a low FRET state with a FRET efficiency of 0.25 and a high FRET state with a FRET efficiency ∼0.78). Complexes without a detectable fluorescence signal from the labeled Mot1 mainly populated the low FRET state as observed for DNA/TBP complexes. The fact that the high FRET peak (*E* = 0.75) is hardly visible in the absence of labeled Mot1 molecules even though most DNA/TBP/Mot1 complexes are in the high FRET state (Figure 1D and E) clearly indicates a high degree of labeling for Mot1. After ATP addition, the relative number of Mot1 containing complexes in the high FRET conformation was reduced and the number of complexes in the low FRET state increased clearly indicating that Mot1 is still bound in the low FRET state after ATP addition. Thus, ATP addition did not liberate Mot1 nor did it cause efficient dissociation of the DNA/TBP complex despite the major conformational change induced by Mot1 binding.

To determine whether ATP binding or ATP hydrolysis is responsible for the conformational change in the DNA/TBP/Mot1 complex, we performed experiments with a non-hydrolysable ATP analog, ATPγS. Similar to ATP addition, the addition of ATPγS almost completely restored the low FRET population (Figure 1E). A very minor fraction of molecules were still observed in the high-FRET state after addition of ATPγS, which could be due to the lower binding affinity of ATPγS to the Mot1-TBP-DNA complex. This population totally disappears after sequential addition of ATP (Supplementary Table S1). The binding of ATP to Mot1 was thus sufficient to induce a conformational change of the DNA/TBP/Mot1 complex from a high FRET state to a low FRET state with a similar FRET efficiency as the binary DNA/TBP complex. However, the same low FRET efficiency does not necessarily imply that the binary and ternary complex upon ATP hydrolysis adopt the same overall DNA/TBP structure.

To rule out any influences of the surface on the conformation and functionality of the DNA/TBP/Mot1 complex, spFRET experiments were also performed in solution using a confocal MFD microscope with pulsed interleaved excitation (57). In addition, the confocal-based measurements can provide information regarding dynamics on the micro- to millisecond timescale, giving insights into the flexibility of the structure. Similar to the TIRFM experiments, DNA/TBP complexes displayed a low FRET efficiency of 0.24, easily observable in the 1D projection, and the same increase in FRET efficiency was observed upon addition of Mot1 in solution (Supplementary Figure S6A-B). The MFD-PIE experiments also clearly indicate that the FRET increase upon Mot1 binding is due to a change in conformation of the complex, which is also dynamic on the millisecond timescale. By monitoring the fluorescence lifetime of the acceptor with direct excitation, we can rule out that the high-FRET state is due to purely Mot1-induced changes in the photophysical properties of the fluorophores (Supplementary Figure S6C-D). Furthermore, complexes with high FRET efficiency completely vanished upon addition of ATP or ATPγS and the majority of complexes adopted a conformation with a FRET efficiency of 0.25 (Supplementary Figure S6E-F) confirming the conformational change observed in TIRF experiments (Figure 1). The low FRET populations fall clearly on the static FRET line, indicating there is no sub-millisecond dynamics in the complexes.

We further investigated the conformational changes between TBP and the DNA induced by Mot1 using the Adenovirus major late promoter (AdML) with an upstream-label (DNA6, Supplementary Figure S1). Interestingly, we observed a similar high FRET efficiency state in the presence of TBP and Mot1 as was observed for the H2B promoter containing DNA (DNA1, Supplementary Figure S7), although with lower amplitude. As the FRET efficiency shifted to higher values in the presence of Mot1 using the AdML promoter labeled upstream (DNA6) as well as for the downstream-labeled H2B promoter (DNA1), we conclude that Mot1 binding induces additional bending of the DNA that brings the fluorophores into close proximity rather than a shift of TBP along the DNA. These findings are supported by DNA footprinting experiments, which show an expansion of the footprint upstream of the TATA box due to Mot1 binding, but no evidence for a shift in TBP position along the DNA (50). The change in FRET efficiency of the ternary complex upon Mot1 binding could, in principle, be induced by a variety of conformational changes within the complex. Mot1 forms contacts with TBP and the DNA, and formation of the ternary complex involves conformational rearrangements of the DNA and/or TBP (29,53,58,59).

### Mot1-induced TBP dissociation from TATA box containing promoters

Above, we monitored FRET between TBP and DNA. Hence, any molecules that exhibited FRET had a TBP molecule bound. The absolute number of TBP-containing complexes (number of complexes on the surface exhibiting FRET) observed in the spFRET experiments did not change upon addition of ATP. These findings imply that ATP binding and hydrolysis by Mot1 is not sufficient to efficiently displace TBP from DNA. This is in striking contrast to what is observed in ensemble experiments with DNA/TBP/Mot1 ternary complexes in solution where ATP addition results in complete clearance of TBP from the DNA (48,53,54). Therefore, we next measured the lifetime of single TBP molecules bound to the TATA box under the influence of Mot1. We used flow chambers that allowed precise control of the concentration of components added to the system. DNA/TBP or DNA/TBP/Mot1 complexes were preformed (using DNA2, Supplementary Figure S1) and immobilized on the prism surface, while non-immobilized complexes were removed from the sample chamber. As DNA/TBP and DNA/TBP/Mot1 complexes are stable on the minute timescale in the absence of ATP (35,60–62), disappearance of the red TBP fluorescence was attributed to photobleaching and the survival time of the TBP signal was used to characterize the rate of photobleaching and dissociation under the experimental conditions. As photobleaching depends on laser power and is thus sensitive to changes in alignment, the photobleaching rate was determined individually for each experiment. When dissociation occurs, complexes thus disappear faster with rates equal to the sum of the rates of photobleaching and dissociation.

**Figure 2.**
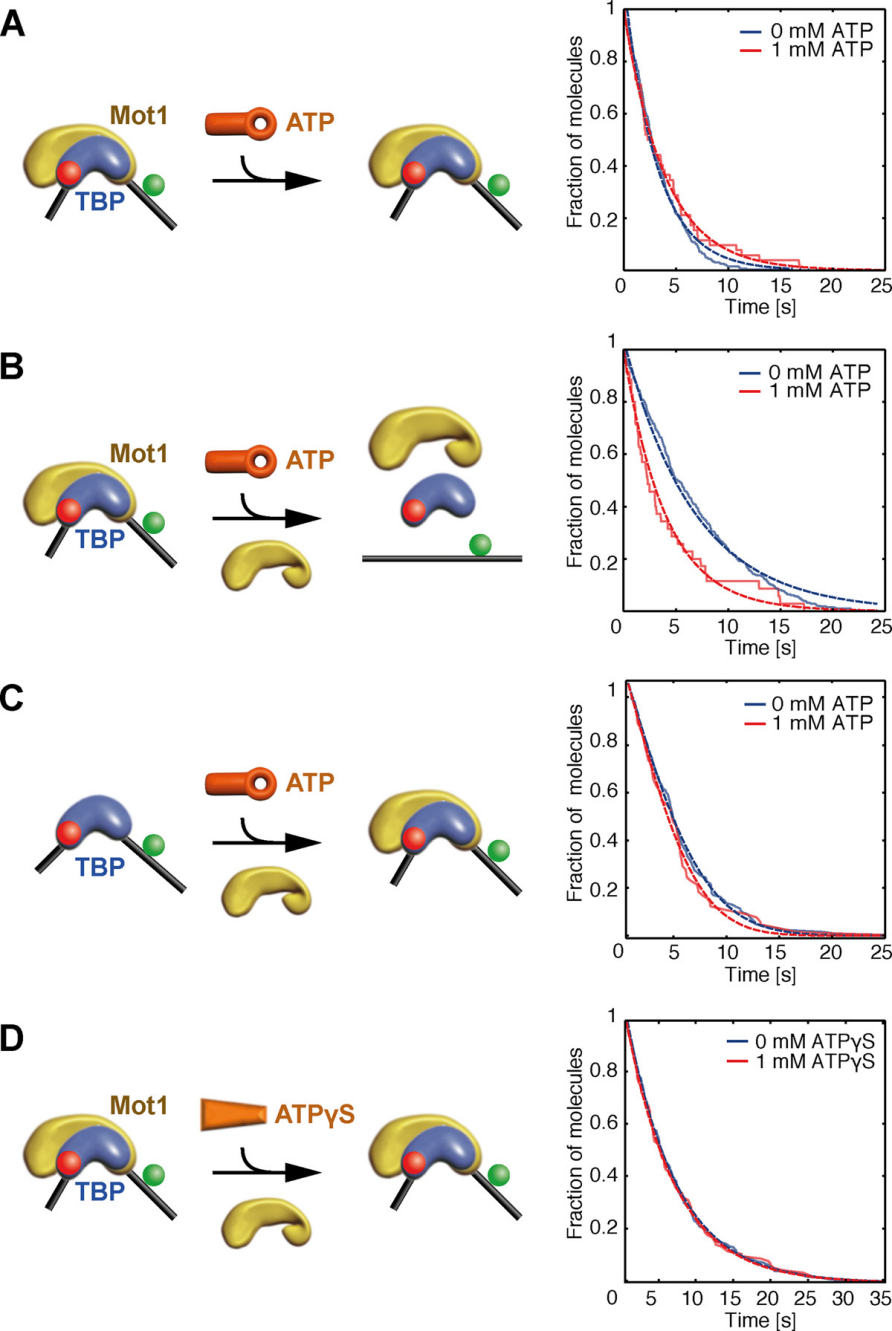
Kinetics of Mot1-catalyzed dissociation of TBP from the H2B promoter (DNA 2). (**A–D**) Time-resolved histograms of detected complexes: number of fluorescent TBP molecules observed over the course of the measurement without ATP addition due to photobleaching (blue) or due to dissociation and photobleaching after addition of 1 mM ATP (red). (A) Addition of 1 mM ATP to pre-assembled DNA/TBP/Mot1 ternary complexes. (B) Combined addition of 1 mM ATP and 3.4 nM Mot1 to preformed DNA/TBP/Mot1 ternary complexes. (C) Addition of 1 mM ATP and 3.4 nM Mot1 to DNA/TBP binary complexes. (D) Combined addition of 1 mM ATPγS and 3.4 nM Mot1 to DNA/TBP/Mot1 ternary complexes. Results of the fits are shown in Supplementary Table S4.

**Figure 3.**
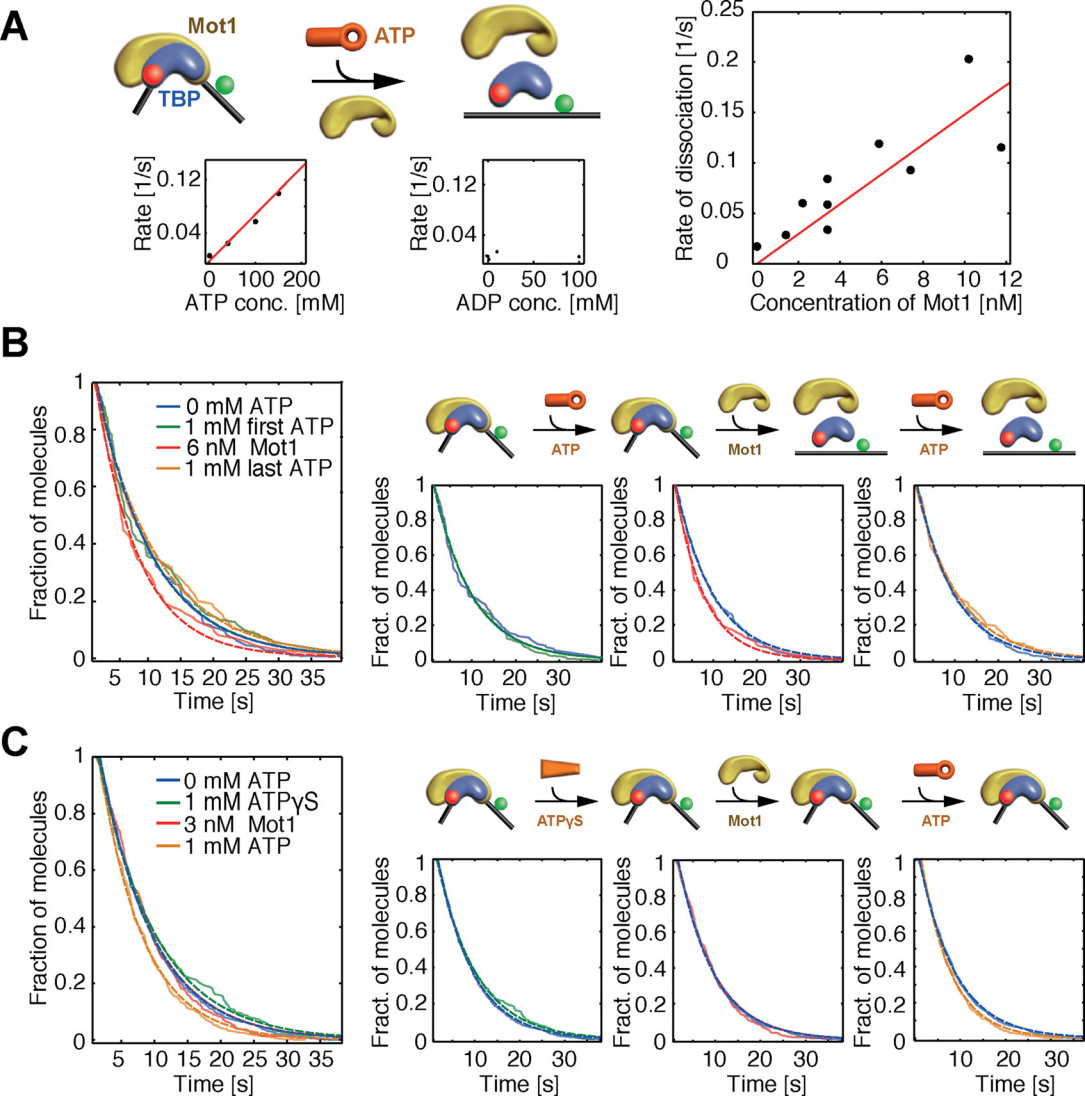
Mot1- and ATP hydrolysis-dependent dissociation of TBP from the H2B promoter (DNA 2). (**A**) The rate of TBP dissociation corrected for photobleaching as a function of ATP concentration (left panel), ADP concentration (middle panel), and Mot1 concentration (right panel) when Mot1 and ATP/ADP were added to immobilized DNA/TBP/Mot1 ternary complexes. (**B**) Sequential addition of ATP and Mot1 to DNA/TBP/Mot1 ternary complexes. The dwell-time histograms of DNA/TBP complexes under all conditions are shown in the left panel. Histograms of the normalized number of fluorescently labeled TBP molecules after addition of 1 mM ATP (second from left), sequential addition of 6 nM Mot1 (second from right) and, after washing out free Mot1, new addition of 1 mM ATP (right panel). (**C**) Sequential addition of ATPγS, Mot1, and ATP to DNA/TBP/Mot1 complexes. The dwell-time histogram of DNA/TBP complexes under all conditions is shown on the left. Histograms of TBP dissociation and photobleaching after addition of 1 mM ATPγS (second from left), sequential addition of 3 nM Mot1 (second from right) and, after removal of Mot1, addition of 1 mM ATP (right panel). Results of the fits are displayed in Supplementary Tables S4 and S5.

The rate of TBP dissociation from DNA was measured under different conditions (Figure 2). For pre-assembled ternary DNA/TBP/Mot1 complexes, no change in survival probability was observed upon addition of ATP in solution (Figure 2A). The lack of TBP dissociation due to insufficient Mot1 binding during the pre-incubation can be ruled out since Mot1 readily binds the DNA/TBP complex as shown in Figure 1D, where the majority of molecules underwent a conformational change upon Mot1 pre-incubation. This is consistent with results from spFRET experiments showing that the ATPase activity of a single Mot1 molecule bound to the DNA/TBP complex did not rapidly dissociate TBP from the TATA box. When both free Mot1 in solution and ATP were added to DNA/TBP/Mot1 complexes, dissociation of TBP from the DNA was clearly visible (Figure 2B). The addition of 1 mM ATP and 3.4 nM Mot1 in solution led to a dissociation rate of ∼0.05/s (Figures 2B and 3A; Supplementary Table S4). Further experiments showed that dissociation of TBP from DNA/TBP binary complexes was not induced by the addition of free Mot1 and ATP, nor could TBP dissociation be observed from DNA/TBP/Mot1 ternary complexes upon addition of free Mot1 in combination with ATPγS (Figure 2C and D). Ternary complex formation was performed at high concentrations before DNA/TBP/Mot1 complexes were diluted to picomolar concentrations for imaging. When Mot1 was added in solution only after DNA/TBP complex formation at high concentrations, the formation of a DNA/TBP/Mot1 complex was not efficient. The results suggest that dissociation of TBP from the TATA box requires one Mot1 molecule bound to the DNA/TBP complex, ATP, and at least a second Mot1 molecule in solution as proposed in Heiss (2011) and Viswanathan *et al.* (2016) (44,63). The experiments with ATPγS indicate that ATP hydrolysis rather than ATP binding is important for dissociation of TBP from DNA. We also observed that the rate of TBP dissociation was dependent on both the concentration of ATP and Mot1, ranging between rates of 0.02/s for *c*(Mot1) = 1 nM and 0.2/s for *c*(Mot1) = 12 nM, and no dissociation was observed when ADP was added in solution with Mot1 (Figure 3A). This gives strong evidence that both ATP and a second Mot1 molecule are needed to dissociate TBP from the H2B promoter. We confirmed these results with ensemble measurements (Section ‘Results R1’ in the Supplementary Data and Supplementary Figure S8).

While prior ensemble measurements suggested the requirement for more than one molecule of Mot1 in the dissociation reaction, the role of ATP hydrolysis by these molecules had not been explored. For this reason, we performed experiments where nucleotides and cofactors were added to the immobilized complexes sequentially (Figure 3B and C; Supplementary Tables S4–5). In a first step, we added ATP to preformed DNA/TBP/Mot1 complexes. This addition of ATP was insufficient to remove TBP from the DNA (Figure 3B, inset left) as observed previously (Figure 2A). After removal of free ATP in solution, Mot1 was added to the chamber and TBP dissociation was observed (Figure 3B, inset middle). This supports our observation that a second Mot1 molecule is needed for TBP dissociation and that the interaction of ATP with the Mot1 in the ternary complex is critical for dissociation of TBP. Removal of the freely diffusing Mot1 and a further round of ATP addition did not lead to further dissociation. When ATPγS was added in the first step instead of ATP, TBP did not dissociate from the DNA (Figure 3C), indicating that ATP hydrolysis and not merely binding is important in the first step. These results provide strong support for the hypothesis that Mot1 bound to the DNA/TBP/Mot1 complex must hydrolyze ATP in order to induce TBP dissociation in a second step that utilizes another molecule of Mot1. Neither ATP binding to the ternary complex nor ATP hydrolysis by Mot1 in solution alone are sufficient for TBP dissociation.

Thus far, the results indicate that a second Mot1 molecule in solution is required for dissociation of TBP from the DNA/TBP/Mot1 complex and that ATP is only required by the Mot1 molecule bound to the ternary complex. This raises the question of whether the molecule in solution needs to be Mot1, or whether a different interaction partner that alters the DNA/TBP interaction can induce TBP dissociation from a complex first primed by Mot1. To test this hypothesis, we performed experiments in which NC2, PC4 or TBP were added in solution along with ATP to ternary DNA/TBP/Mot1 complexes. Dissociation of TBP from the DNA/TBP/Mot1 complex was not observed under these conditions (Supplementary Figure S9A). We did not observe dissociation upon addition of unlabeled DNA with (DNA 4) or without a TATA box (DNA 5) (Supplementary Figure S9B-D; Supplementary Figure S1). These findings support specific functional roles for Mot1 in both the ternary complex and in solution.

### Effect of Mot1 binding on the DNA/TBP conformation

**Figure 4.**
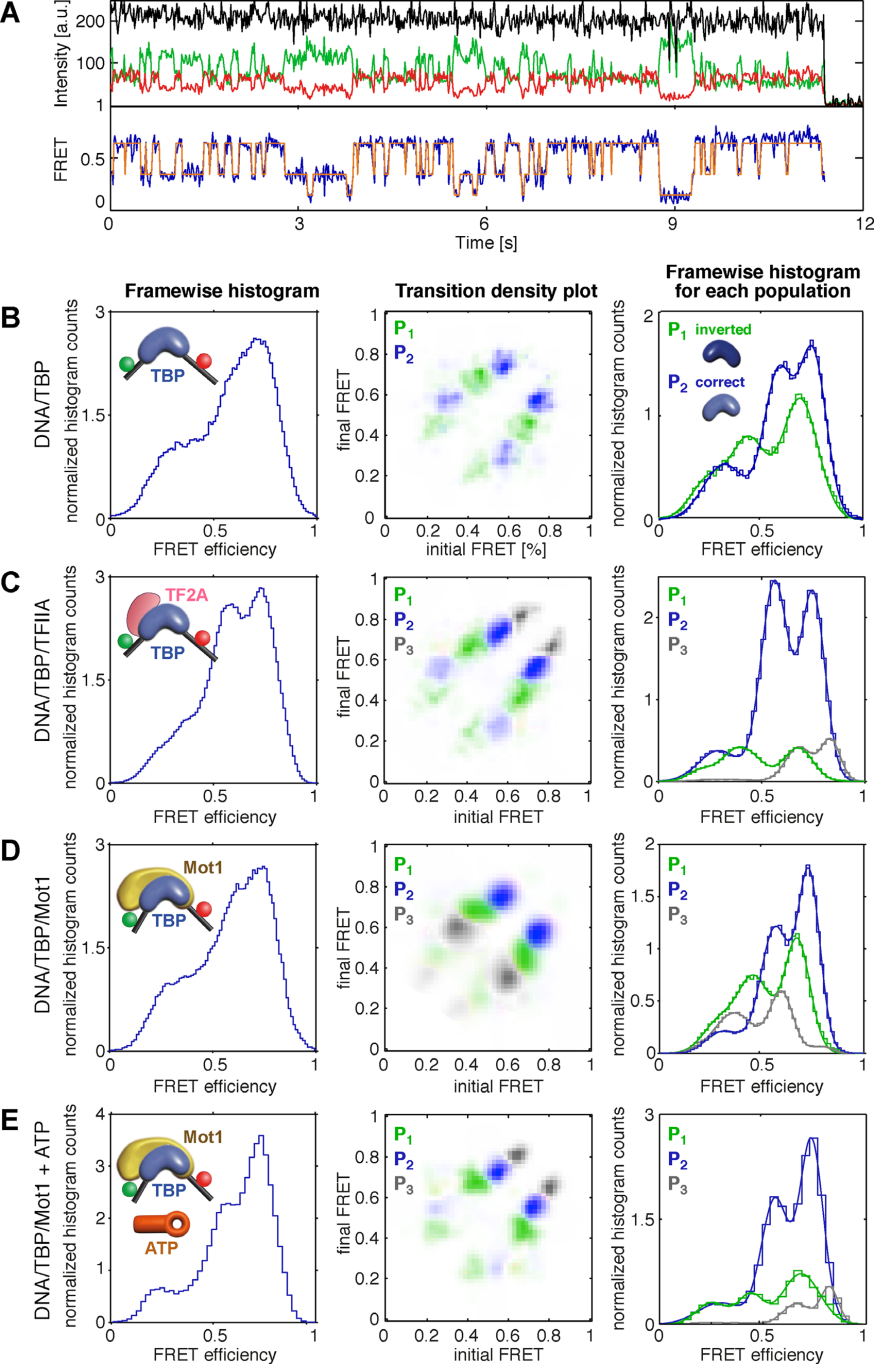
TBP induced conformational dynamics of a dual-labeled H2B promoter (DNA 3) observed by changes in FRET efficiency. (**A**) An exemplary time trace of a dynamic DNA/TBP complex. Donor and acceptor fluorescence are shown in green and red, respectively. The *γ*-corrected total intensity, scaled by a factor of 1.6 for clarity, is displayed in black. The FRET efficiency is shown in blue. The most likely sequence of FRET states obtained by training an HMM model is shown in red. (**B–E**) Normalized frame-wise FRET efficiency histograms of all dynamic DNA molecules (left column) including a schematic representation of the measured complex (inset), TDP of dynamic complexes with color coded subpopulations (middle column) and normalized frame-wise FRET efficiency histograms of the individual subpopulations (right column) for (B) DNA/TBP complexes, (C) DNA/TBP/TFIIA complexes, (D) DNA/TBP/Mot1 complexes, and (E) DNA/TBP/Mot1 complexes upon addition of 1 mM ATP. The number of molecules in each experiment is given in Supplementary Table S1. Different subpopulations of molecules (P_1_, P_2_, and P_3_) are displayed in the TDP and frame-wise histogram in green, blue and gray, respectively.

To elucidate the conformation of the promoter DNA upon complex formation with TBP/Mot1, we formed complexes with dual-labeled DNA constructs (DNA 3, Supplementary Figure S1) that have a FRET pair flanking the TBP-binding sequence. In this experimental set-up, binding of TBP to the DNA is detected as a change in FRET efficiency between the two fluorophores whose proximity increases as a result of DNA bending induced by TBP binding. To investigate potential photophysical artifacts from protein binding to fluorophores labeled on the DNA, MFD-PIE experiments were performed (Supplementary Figure S10) and two different FRET pairs were compared (Supplementary Figure S11). The different dye pairs yielded similar results and no photo-induced artifacts were observed. In the TIRF data, two types of TBP-containing complexes were observed. Around 50% of the molecules exhibited static FRET indistinguishable from the static FRET values observed from DNA alone (Supplementary Figure S12). Based on previously published work (64), these static DNAs in the low FRET state most likely do not have a TBP bound. The remaining ∼50% of the molecules showed a highly dynamic FRET signature with anti-correlated donor and acceptor intensities and a broad distribution of FRET values. An exemplary time trace of a TBP-bound dynamic DNA is displayed in Figure 4A. The corresponding frame-wise histogram of the FRET efficiencies obtained for all dynamic DNA complexes is shown in Figure 4B, left panel. Using HMM analysis (65), three distinct FRET states were determined. The 2D transition density plot (TDP) displaying the initial and final FRET states of each transition given by the HMM analysis on all dynamic DNA complexes is shown in Figure 4B (middle panel). It reveals six distinct FRET populations (or eight clusters, as the intermediate FRET state can transition to both the higher and lower FRET states) that emerged when all transitions of the dynamic complexes were plotted. Based on the TDP, individual clusters were selected and underlying molecules identified and segmented into two subpopulations. The subpopulations (P_1_ and P_2_) are shown in green and blue, respectively (Figure 4B, middle and right panel). Within each subpopulation of molecules (P_i_), three distinct FRET states were occupied and no interconversion between subpopulations was observed during the time of the experiment. Each of the subpopulations thus shows transitions between three distinct, but subpopulation-wise distinct FRET states (S_i_). The FRET values of each subpopulation were labeled with increasing FRET efficiency from S_1_, for the state with lowest FRET efficiency, to the intermediate state S_2_, and the state with highest FRET efficiency, S_3_ (Table 2). The difference between the two subpopulations in terms of FRET values is that the three FRET states for each subpopulation are shifted with respect to each other (Table 2). Interestingly, the TDP indicates that the three states are connected via a linear three-well model. Thus, a transition between S_1_ and S_3_ can only occur via S_2_. Interestingly, the FRET efficiency of the low FRET conformation is less than what is expected for double-helix DNA alone. This suggests an untwisting or expansion of the DNA equivalent to the displacement by one additional base (over the donor-acceptor separation of 18 bases) leading to an increased separation of the donor and acceptor molecules.

**Table 2.** Parameters of the normal distributions obtained by fitting the frame-wise FRET efficiency histograms shown in Figure 4

	S_1_			S_2_			S_3_		
	A_1_	μ_1_	σ_1_	A_2_	μ_2_	σ_2_	A_3_	μ_3_	σ_3_
DNA/TBP									
P_1_ (43.5%)	18	25	9	51	44	8	31	69	9
P_2_ (56.4%)	21	32	10	39	59	7	40	76	7
DNA/TBP/TFIIA									
P_1_ (18.3% / 16.2%)	9	19	6	53	41	9	39	68	7
P_2_ (81.7% / 72.2%)	11	29	10	48	56	7	41	75	6
P_3_ (—-/ 11.7%)	6	32	12	51	68	7	43	83	5
DNA/TBP/Mot1									
P_1_ (41.8% / 34.4%)	15	25	8	39	45	9	46	69	7
P_2_ (58.2% / 47.9%)	10	30	10	43	57	8	47	76	6
P_3_ (— / 17.8%)	51	35	11	44	61	6	5	81	6
DNA/TBP/Mot1+ATP									
P_1_ (23.1% / 20.2%)	23	25	7	30	45	7	47	68	7
P_2_ (77.0% / 67.5%)	11	30	11	39	56	6	50	74	6
P_3_ (— / 12.4%)	15	32	14	42	67	7	43	82	4

The mean FRET efficiency μ and standard deviation σ are given as FRET efficiency (in %). The relative areas of the Gaussian distribution A is given in % for each subpopulation. The ratios of the area-weighted contributions of each population compared between two or three fractions respectively are given in %.

The two subpopulations of DNA detected upon TBP binding might be the result of bidirectional binding of TBP to the TATA-box sequence (66,67). To test this hypothesis, we added the TFIIA to the binary complex, which is known to stabilize TBP orientation-specifically on the DNA (66,67) (Figure 4C). In previously published spFRET measurements (56), we showed that the presence of TFIIA shifted the orientation of TBP on DNA containing the AdML promoter with similar effectiveness as was measured by Cox *et al.* (66). For the current experiments with the H2B promoter, the relative number of complexes observed in the P_2_ subpopulation increased from 51% for the DNA/TBP complex to 81% after pre-incubation with TFIIA. Subpopulation P_1_, on the other hand, decreases in the presence of TFIIA. The results of the experiments strongly suggest that the DNA molecules belonging to subpopulation P_2_ are complexes where TBP is bound in the preferred orientation with respect to the direction of transcription (67,68). Upon addition of TFIIA, a third subpopulation was also observed that could not be clearly assigned to subpopulation P_1_ or P_2_ and hence we assigned it to a new subpopulation, P_3_. This subpopulation is potentially due to the influence of TFIIA on the photophysical properties of the donor or a different conformation of the complex. However, as the subpopulation is only a minor species, we concentrated on the ratio of subpopulations P_1_ and P_2_ in further experiments.

We also investigated the dynamics of the DNA in the DNA/TBP/Mot1 complex. Similar to what we observed in the presence of TFIIA, three subpopulations, P_1_, P_2_ and P_3_, were detected, each of which interconverted between three FRET states (Figure 4D and Table 2). Again, the fraction of complexes assigned to P_3_ was minor and the ratio of the subpopulations P_1_ and P_2_ stayed constant upon addition of Mot1, suggesting that Mot1 binds to TBP irrespective of its orientation. Upon addition of ATP to the ternary complexes, subpopulation P_2_ was observed with a higher frequency compared to the other subpopulations and the spFRET histogram looks very similar to that in the presence of TFIIA (Figure 4C and E). This suggests that Mot1, in the presence of ATP, leads to a redistribution of TBP on the DNA by preferential dissociation of Mot1 bound in the incorrect orientation.

Interestingly, no reorientation of TBP on the DNA was observed during the time of the experiment (DNA/TBP complexes were observed on average for 5.8 s). Neither TFIIA nor Mot1 were observed to directly invert the binding orientation of TBP on the DNA; rather, this finding supports a model in which the inversion of the TBP binding orientation requires TBP dissociation and rebinding. The increased number of DNA/TBP complexes with the preferred binding orientation in the presence of TFIIA or Mot1-ATP can be explained by the modulation of the stability or the binding affinity of the binary complex by these cofactors.

## DISCUSSION

**Figure 5.**
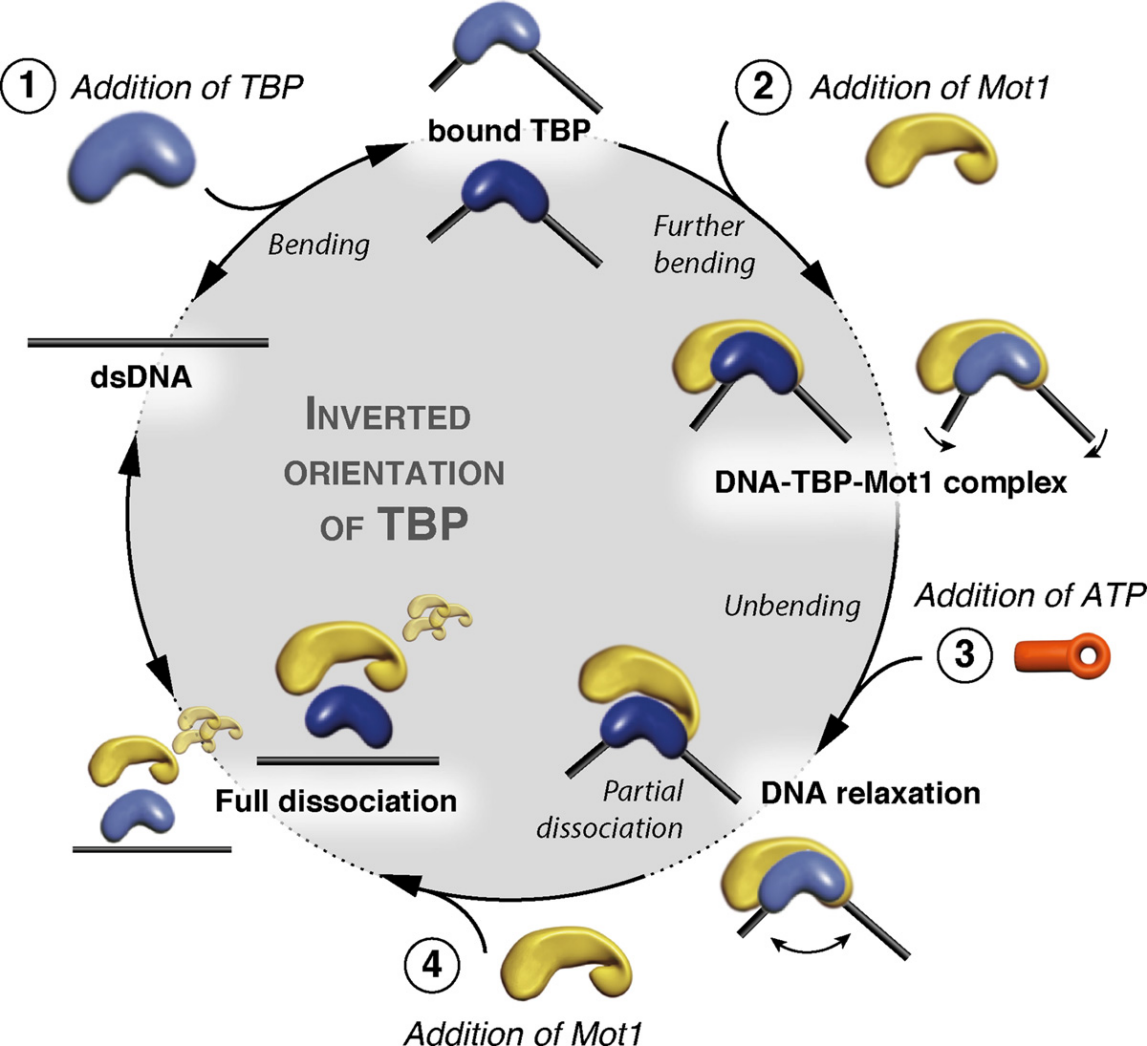
Scheme of the TBP/DNA conformational cycle upon Mot1 and ATP addition. Step 1: TBP (blue) binds to dsDNA and induces a bent state. Binding of TBP to the TATA box can occur in two orientations. TBP is depicted in light blue when bound correctly (outer circle), and depicted in dark blue when bound in the inverted orientation (inner circle, light gray background). Step 2: Mot1 (yellow) binds to the binary TBP/DNA complex and forms a stable ternary TBP/DNA/Mot1 complex. This binding step induces an additional bending and conformational change in the TBP/DNA interactions. Step 3: Addition of ATP leads to partial dissociation and ‘primes’ the complex in an unbent DNA conformation. Complexes where TBP is bound in the inverted binding orientation are the primary target for priming (dark blue/yellow, inner circle). Step 4: Addition of additional Mot1 in solution together with ATP liberates TBP from the primed TBP/DNA complex.

As DNA/TBP complexes are relatively stable with lifetimes of 15 min to 1 h (35,60–62), the regulation of complex formation and stability by additional cofactors is highly important to adapt transcription initiation to the cellular conditions. Here, we investigated the molecular mechanism of Mot1 on stably formed yeast DNA/TBP complexes using spFRET by analyzing conformational transitions of DNA/TBP complexes upon Mot1 binding and ATP hydrolysis. We found that ATP addition to ternary DNA/TBP/Mot1 complexes was not sufficient to dissociate TBP from the DNA. These results are consistent with recent studies on Mot1 function implicating that it mainly removes weakly bound TBP (44,45). *In vivo*, the inefficiency in Mot1 action in removing stably bound TBP may allow for competition between Mot1 and other TBP-binding factors that promote formation of the transcription PIC. Several models have been proposed for the dissociation of TBP from the TATA box by Mot1, including direct displacement of TBP by translocation of Mot1 along the DNA (48,50), indirect displacement by modulations of the interactions of TBP with DNA (58), or a combination of both (29,30,53). These models explain the dissociation of TBP from the DNA and thus the repression of gene expression by Mot1. As a general framework for how Mot1 is able to up-regulate gene expression, it was proposed that Mot1’s ATPase activity liberates transcriptionally inactive TBP and, in turn, redistributes TBP along the DNA (42,69,70). The model proposed by Sprouse *et al.* (62) is in agreement with results in this work explaining the role of Mot1 in gene activation by selectively dissociating TBP bound in the inverted binding orientation in a two-step process. In a first step, Mot1 introduces a conformational change in the DNA/TBP complex. Second, TBP is dissociated from the DNA in the presence of additional Mot1 as previously suggested by Moyle-Heyrman *et al.* (59), while ATP hydrolysis is performed by Mot1 bound to the complex (Figure 5). Two scenarios for the induction of ATP hydrolysis by Mot1 in solution could be imagined. First, ATP hydrolysis by Mot1 might be performed directly upon ATP binding to the DNA/TBP/Mot1 complex, priming the complex for dissociation by proximity to an additional Mot1 molecule. A second scenario involves a DNA/TBP/Mot1 complex with bound ATP, where freely diffusing Mot1 is necessary for the induction of ATP hydrolysis by Mot1 bound to the complex. In either case, the α-helical latch loops of Mot1 may be involved in the dissociation of TBP from the DNA as they interact with the DNA-binding region of TBP (29). If the α-helical latch of the freely diffusing Mot1 molecule were to replace the DNA in the DNA binding region, the TBP could then easily dissociate from the DNA and remain together upon DNA dissociation with the Mot1 molecule (36). In *mot1* cells, TBP occupancy at selected promoters increased but the occupancies of TFIIB and Pol II did not, leading to the suggestion that Mot1 displaces TBP complexes that are not assembled into transcriptionally competent complexes (45,69). At the yeast *URA1* promoter, which harbors a naturally occurring TATA box that preferentially binds TBP in the reverse orientation with respect to transcription, Mot1 is responsible for TBP displacement but removal of incorrectly oriented TBP cannot fully explain Mot1’s role in *URA1* activation (62). TFIIB binding can be influenced by promoter sequence (71,72) and while it has not been demonstrated to be generally true, it appears likely that TFIIB is in general excluded from incorrectly oriented TBP.

Using dual-labeled DNA (Figure 4), two different subpopulations of DNA/TBP complexes were observed. In agreement with previous studies (66,67), these could be attributed to TBP binding to the TATA box in both orientations. TFIIA is known to stabilize the transcription-competent orientation of TBP (66–68) and it clearly shifted the ratio of both DNA/TBP subpopulations. In the presence of Mot1 and ATP, we observed similar effects on the DNA/TBP complex as for TFIIA. This suggests that the subpopulation of molecules with TBP bound in the transcriptionally active orientation is strongly favored over the second subpopulation. Mot1 therefore has a previously unsuspected role in stabilizing correctly-bound TBP on the DNA, while it specifically dissociates TBP bound in the opposite orientation. This is consistent with evidence suggesting that Mot1 has a role in dissociating incorrectly oriented TBP on a natural yeast promoter (62). Mot1’s ability to displace incorrectly oriented TBP can also explain its activating effect on transcription. This observation is consistent with recent studies that provided evidence that Mot1 is mainly responsible for TBP dissociation from low affinity sites *in vivo* (45) and is less efficient at dissociating DNA/TBP complexes with high affinity interactions (44).

It is interesting that static FRET signals were observed when monitoring the signal between labeled TBP and DNA while dynamic FRET signals are visualized when monitoring different positions on the DNA. One explanation for this apparent discrepancy is that the label on the TBP is located near the hinge on the DNA and as such is not sensitive to the conformational changes in the DNA. A second possibility is that placing the label at position −6 on the H2B-promoter-containing DNA destabilized the TBP/DNA complex, leading to fluctuations. Regardless of the origin of the dynamics, we can use the sensitivity of the FRET labels to the dynamics to investigate the influence of the different transcription factors on the stability of the complex.

Taking advantage of the single-pair FRET approach, we have elucidated the role of Mot1 on the conformation of the DNA/TBP complex and dissociation of TBP. Mot1’s dissociating activity depends on ATP hydrolysis in the DNA/TBP/Mot1 complex and additional Mot1 molecules in solution. In addition, Mot1 shows a TBP-orientation specific activity, suggesting that regulation of TBP binding orientation is important for the regulation of gene expression *in vivo*.

## Supplementary Material

Supplementary Data
