# Supplementary material for: Conformational changes and catalytic inefficiency associated with Mot1-mediated TBP–DNA dissociation
Source: Nucleic Acids Res. 2019 Jan 15;47(6):2793–806. doi: 10.1093/nar/gky1322 (PMC6451094; doi:10.1093/nar/gky1322)
Supplement: Supplementary Data [file gky1322_supplemental_files.pdf]

Supplementary Information

Conformational changes and catalytic inefficiency associated with Mot1-mediated TBP-DNA dissociation.

Gregor Heiss[†], Evelyn Ploetz[†], Lena Voith von Voithenberg[†], Ramya Viswanathan, Samson Glaser, Peter Schluesche, Sushi Madhira, Michael Meisterernst, David Auble, Don C. Lamb

Page

Supplementary Material and Methods............................. 2

Supplementary Results... 4

Supplementary Figures
- Supplementary Figure S1..................................... 5
- Supplementary Figure S2..................................... 6
- Supplementary Figure S3..................................... 8
- Supplementary Figure S4..................................... 9
- Supplementary Figure S5..................................... 10
- Supplementary Figure S6..................................... 11
- Supplementary Figure S7..................................... 13
- Supplementary Figure S8..................................... 14
- Supplementary Figure S9..................................... 15
- Supplementary Figure S10................................... 16
- Supplementary Figure S11................................... 17
- Supplementary Figure S12................................... 19

Supplementary Tables
- Supplementary Table S1....................................... 20
- Supplementary Table S2....................................... 21
- Supplementary Table S3....................................... 22
- Supplementary Table S4....................................... 23
- Supplementary Table S5....................................... 24

References... 25

Supplementary Material and Methods

Determination of number of states in HMM.

The robustness of the Hidden Markov analysis allows one to model different types of processes in an unsupervised manner for independent observation sequences. To determine the correct number of involved (hidden) states via HMM, we applied the Akaike information criterion (AIC) and Bayesian information criterion (BIC) using the following formulas, respectively:

$$\mathrm{AIC} = -\,2\cdot\log(L) + 2\cdot p$$
$$\mathrm{BIC} = -2\cdot\log(L) + p\cdot\log(N) \qquad \text{Eqn. S1}$$

L describes the likelihood function of the model, N the number of observation points, and p the number of estimated parameters within the model. We assume that the FRET signal associated with certain conformations of DNA can be described by a Gaussian distribution, while modelling the interaction of TBP with double-stranded DNA in the presence of different co-factors. We describe the Gaussian distribution by a parameter set of $k = 2$, which refers to the mean μ and width σ. Assuming a number of m hidden states, the number of independent parameters p, is given as

$$p = m^2 + k\cdot m - 1 \qquad \text{Eqn. S2}$$

While the log likelihood always increases with the incorporation of more states, the AIC and BIC should show a minimum at the optimal number of states. **Supplementary Figure S3** shows the log likelihood, AIC and BIC criteria when varying the number of states for the in the model from 1 to 10 for the data shown in Figure 4. Instead of giving an optimal number of states, both the AIC and BIC follow a monotony decreasing behaviour that plateaus with three states in the model.

Structural modelling and accessible volume calculation.

To visualize the TBP/Mot1 protein complex on bent DNA as well as the position of attached fluorophores, we started with the crystal structure of the preinitiation complex published by Kornberg et al (Ref. (1), pdb 5FMF). The Mot1 structure was published by Butryn et al. (Ref. (2), pdb 4WZS). We imported both pdb files in PyMOL (3) and aligned the structures using the TATA-binding protein. After removing all additional TFs that were co-crystallized in the structure, we modelled the presented DNA based on the employed sequence for DNA1. The bent DNA was modelled and exported as pdb using 3D-DART (4). The DNA structure was afterwards aligned with the DNA sequence, in particular the TATA-sequence of the DNA fragment that was co-crystalized in the Kornberg structure (pdb 5FMF). Afterwards we removed all other DNA fragments from the structure and saved the resulting structure of bent DNA/TBP/Mot1 as a new pdb file. Next, starting from the new pdb file, we determined the ID of atoms to which the fluorophores, i.e. Atto532 and Atto647n are attached on the DNA respectively TBP. With this knowledge, we determined the accessible volumes (AV) of the dyes (5) on the DNA/TBP/Mot1 structure and displayed the DNA/TBP/Mot1 complex together with the calculated AVs in PyMOL.

Supplementary Results

R1 - Dissociation of TBP from DNA requires ATP and an additional Mot1 in Solution

To directly compare results obtained with single complexes to ensemble results, we tested the activity of Mot1 on complexes assembled on DNA templates tethered to beads via a biotin linkage at one end of one DNA strand (**Supplementary Figure S8A**). Immobilizing the complexes allowed us to assemble them and then remove unbound material by washing as done previously (42). The addition of ATP to preformed, purified DNA/TBP/Mot1 complexes did not induce any detectable release of TBP into the supernatant, while the combined addition of ATP and competitor DNA led to the release of a relatively small amount of TBP (**Supplementary Figure 8B**). More TBP was released from the beads upon addition of ATP, Mot1, and competitor DNA in solution. Competitor DNA was required to observe the release of free TBP in the supernatant (42) as the local concentration of DNA on the beads is high. Thus, dissociated TBP can rebind to a neighboring DNA molecule after release and competitor DNA is required to keep it in solution. In single molecule experiments, the concentration of DNA on the surface is low (pM) and the probability of TBP rebinding to the DNA negligible. In conclusion, Mot1 behaves similarly in both, spFRET experiments and ensemble experiments using immobilized complexes.

Suppl. Figure S1

DNA 1

```
                                                         Atto647N
5’-Biotin-CTT CAC CTT ATT TGC ATA AGC GAT TCT ATA TAA AAG CGC CTT GTC ATA CCC TGC TCA CGC TGT TTT TCC TTT TCG TTG GC-3’
       3’-GAA GTG GAA TAA ACG TAT TCG CTA AGA TAT ATT TTC GCG GAA CAG TAT GGG ACG AGT GCG ACA AAA AGG AAA AGC AAC CG-5’
```

DNA 2

```
                                                         Atto532
5’-Biotin-CTT CAC CTT ATT TGC ATA AGC GAT TCT ATA TAA AAG CGC CTT GTC ATA CCC TGC TCA CGC TGT TTT TCC TTT TCG TTG GC-3’
       3’-GAA GTG GAA TAA ACG TAT TCG CTA AGA TAT ATT TTC GCG GAA CAG TAT GGG ACG AGT GCG ACA AAA AGG AAA AGC AAC CG-5’
```

DNA 3

```
                                                         Atto647N
5’-Biotin-CTT CAC CTT ATT TGC ATA AGC GAT TCT ATA TAA AAG CGC CTC GCT CTA CCC TGC TCA CGC TGT TTT TCC TTT TCG TTG GC-3’
       3’-GAA GTG GAA TAA ACG TAT TCG CTA AGA TAT ATT TTC GCG GAG CGA GAT GGG ACG AGT GCG ACA AAA AGG AAA AGC AAC CG-5’
                                 Atto532, Atto565, Cy3B
```

DNA 4

```
5’-Biotin-CTT CAC CTT ATT TGC ATA AGC GAT TCT ATA TAA AAG CGC CTC GCT CTA CCC TGC TCA CGC TGT TTT TCC TTT TCG TTG GC-3’
       3’-GAA GTG GAA TAA ACG TAT TCG CTA AGA TAT ATT TTC GCG GAG CGA GAT GGG ACG AGT GCG ACA AAA AGG AAA AGC AAC CG-5’
```

DNA 5

```
   5’-GAC CGC GAA TAT TCG CGC GTT ATT TCG ATA GC-3’
   3’-CTG GCG CTT ATA AGC GCG CAA TAA AGC TAT CG-5’
```

DNA 6

```
                              Atto647N
   5’-GCC ACG TGA CCG GGT GTT CCT GAA GGG GGG CTA TAA AAG GGG GTG GGG GCG CGT TCG TCC TCA CTC TCT T-Biotin-3’
   3’-CGC TGC ACT GGC CCA CAA GGA CTT CCC CCC GAT ATT TTC CCC CAC CCC CGC GCA AGC ACC ACT GAG AGA A-5’
```

Figure S1. Oligonucleotides investigated in the presence of TBP. DNAs 1-5 are 80 bp DNA strands containing the H2B promoter site. DNA 1 was labeled with the acceptor fluorophore and was used to analyze the conformation of the DNA/TBP complex. DNA 2 was labeled with the donor fluorophore and was used for TBP dissociation experiments. DNA 3 is dual-labeled and was used to investigate the conformation of the DNA upon protein binding. DNA 4 is an unlabeled version of DNA 1. DNA 5 is a non-promoter DNA missing the TATA box. DNA 6 contains the TATA-box sequence from the adenovirus major late promoter.

Suppl. Figure S2

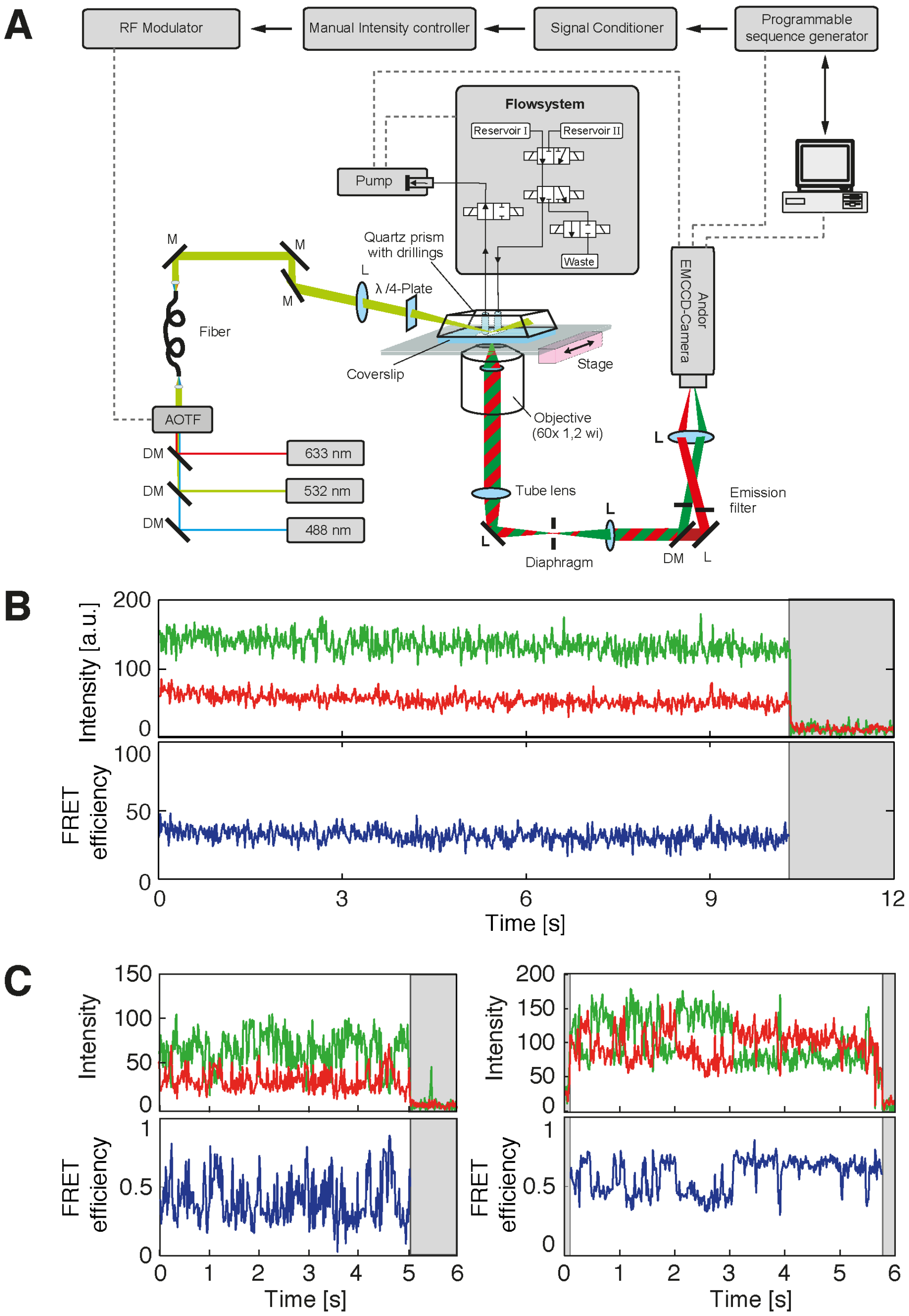

Figure S2. Schematic overview of our prism-type TIRF experiments. (**A**) Schematics of the experimental setup. Different laser lines are coupled through an AOTF into a single mode polarization maintaining fiber. Laser excitation is focused under TIRF conditions on the prism hypotenuse where the sample is immobilized on the surface and fluorescent molecules within the evanescent field are illuminated. Fluorescence is collected by a 60x 1.2 NA water immersion

objective, spectrally separated by dichroic mirrors, and imaged on an Andor EM-CCD camera. The design of the flow chamber allows external connection and control of the flow during data acquisition. (**B**) Static and (**C**) Dynamic single-molecule time traces of DNA/TBP/Mot1 complexes labeled using DNA 1-Atto647N, TBP-Atto532. Upper panel: The donor and acceptor intensities are shown in green and red, respectively. Lower panel: The calculated FRET efficiency is shown in blue.

Suppl. Figure S3

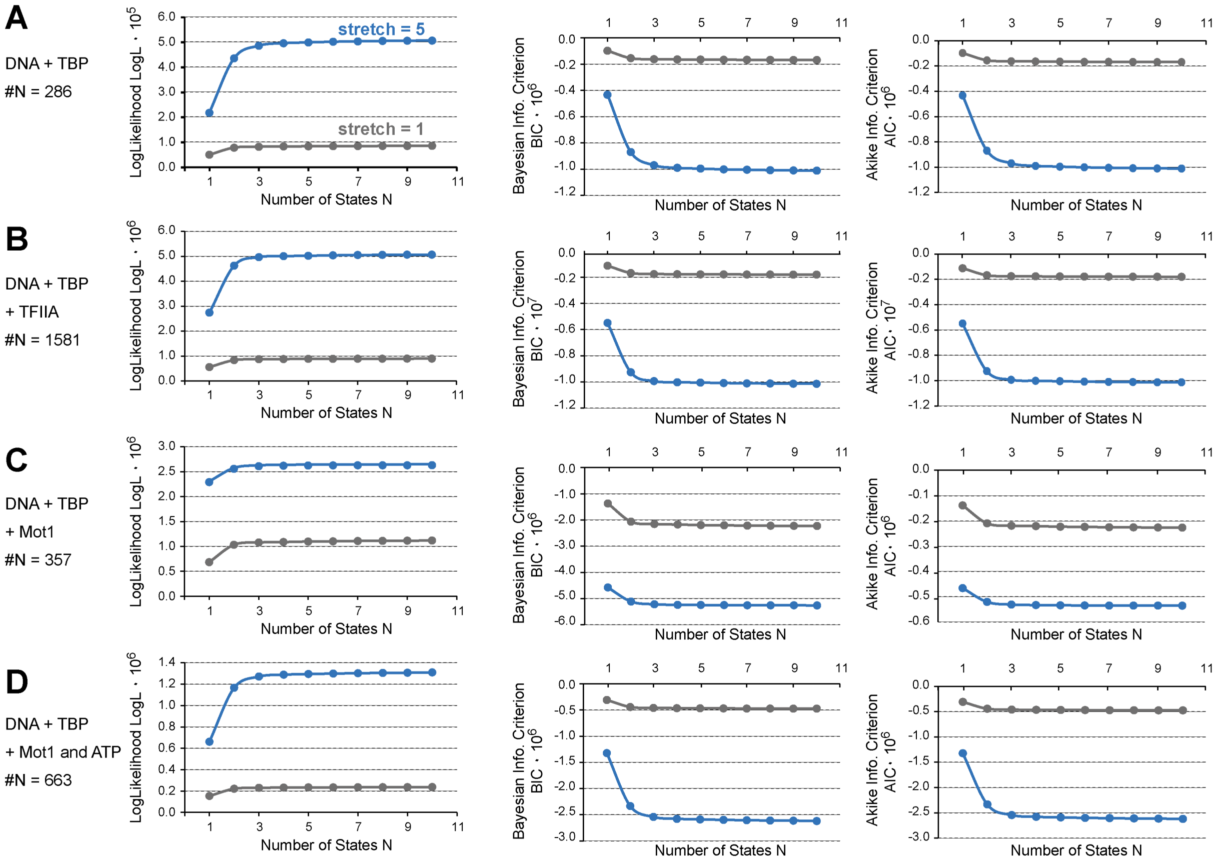

Figure S3. Number of states. (**A-D**) *left panel* The log-likelihood, *middle panel* the AIC and *right panel* the BIC criterion of a local Hidden Markov Model analysis are plotted as a function of the number of states in the model. Results are shown for the spFFRET measurements with dual-labeled H2B promoter DNA in the presence of **(A)** TBP, **(B)** TBP and TFIIA, **(C)** Mot1, as well as **(D)** Mot1 and 1mM ATP as described in Figure 4. HMM fits to the original data are shown in grey and for a stretching factor of 5 (provided in the software to improve the modelling of quick transitions) in blue. 5Start parameters were chosen assuming a self-adapting μ and an initial standard deviation of $\sigma = 0.05$. The number of iterations was limited to a maximum of 10^5. The convergence threshold was set to 10^{-6}.

Suppl. Figure S4

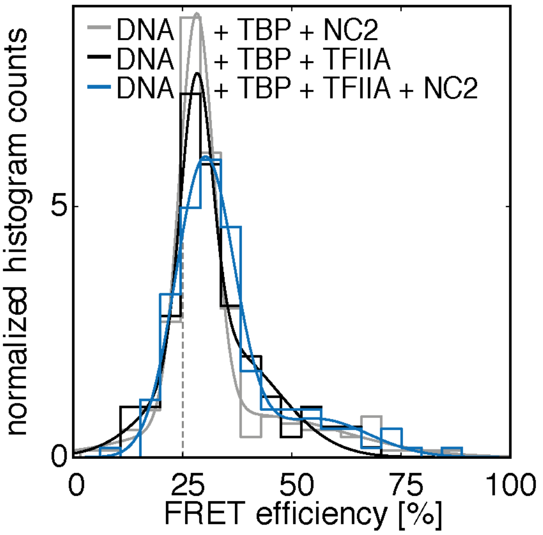

Figure S4. Conformation of the DNA/TBP complex in the presence of TF2A or NC2. Molecule-wise FRET efficiency histogram of 200 static DNA/TBP/NC2 (grey), 134 DNA/TBP/TFIIA (black) and 116 DNA/TBP/TFIIA/NC2 (blue) complexes.

Suppl. Figure S5

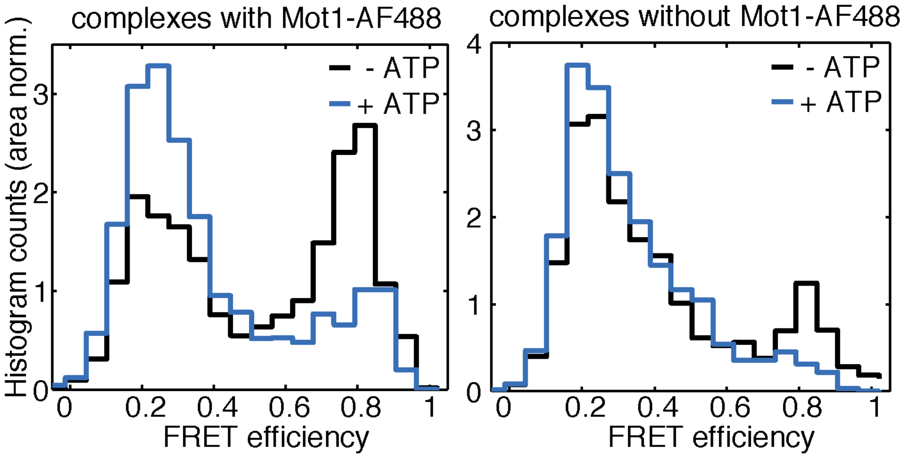

Figure S5. Ternary DNA/TBP/Mot1 complexes in a three-color experiment with DNA 1-Atto647N, TBP-Atto532, and Mot1-Alexa488. Normalized FRET efficiency histogram of frame-wise FRET between DNA and TBP of complexes containing Alexa488-labeled Mot1 (left) and of complexes without detection of Alexa488-labeled Mot1 (right). Black: before addition of ATP. Blue: after addition of 1 mM ATP.

Suppl. Figure S6

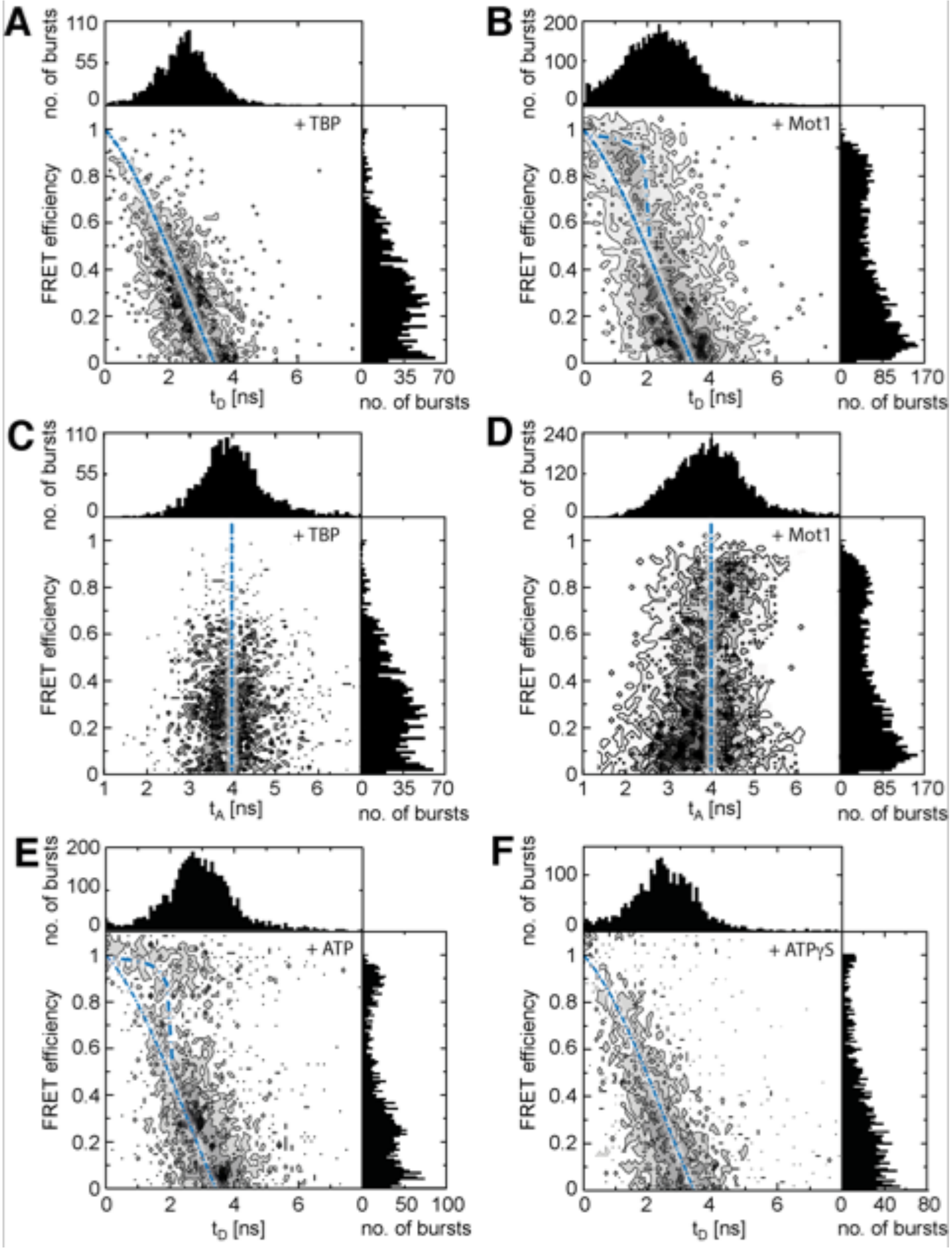

Figure S6 Burst analysis of preincubated DNA/TBP complexes in solution by confocal microscopy. (**A,B**) Two-dimensional histograms of FRET efficiency E versus donor lifetime $\tau_{D[A]}$ for Atto532 fluorescently labeled TBP bound to DNA 1. (**A**) DNA/TBP complex. (**B**) TBP/DNA/Mot1 complex. (**C,D**) Two-dimensional histograms of FRET efficiency versus

acceptor lifetimes τ_A for the experiments plotted in panels A and B. The average lifetime is shown in blue. No significant shift in lifetime is observed in the presence of Mot1. (**E-F**) Two-dimensional histograms of FRET efficiency E versus donor lifetime $\tau_{D[A]}$ for Atto532 fluorescently labeled TBP bound to DNA 1. (**E**) Ternary complex after addition of 1 mM ATP. (**F**) Ternary complex after addition of 1 mM ATPγS. Populations of molecules static in solution during their diffusion time though the focal volume (micro- to milliseconds) are found on the static FRET curve (blue line), a polynomial describing the relationship between the donor lifetime and the FRET efficiency. A deviation to the right of this static FRET line indicates conformational dynamics between different FRET states. Deviations to the left of the line hint at fluorescence quenching.

Suppl. Figure S7

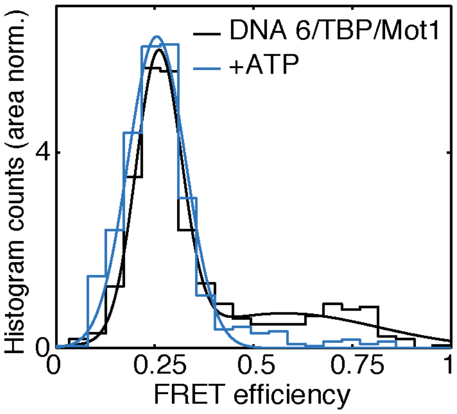

Figure S7. Ternary DNA/TBP/Mot1 complexes on AdML promoter. FRET efficiency histogram of DNA/TBP/Mot1 complexes exhibiting static FRET. The FRET efficiency was measured between TBP and upstream-labeled dsDNA containing the AdML promoter (DNA 6). Upon addition of Mot1, an additional high-FRET population is observed, which diminishes in the presence of ATP.

Suppl. Figure S8

A

5' Bio

```
Bio-GGCCCGGGATCCCCGACCGGGTGTTCCTGAAGGGGGGCTATAAAAGGGGGTGGCGCGTGAATTCGCGC
    CCGGGCCCTAGGGGCTGGCCCACAAGGACTTCCCCCCGATATTTTCCCCCACCGCGCACTTAAGCGCGGG
```

B

**TBP**	+	+	+	+	+	+	+	+	+	+
**Mot1**	+	+	+	-	+	+	+	+	+	+
**ATP**	+	+	+	-	-	-	+	+	+	+
**Mot1 (soln)**	-	+	+	-	-	-	-	-	+	+
**Competitor**	+	-	+	+	-	+	-	+	-	+

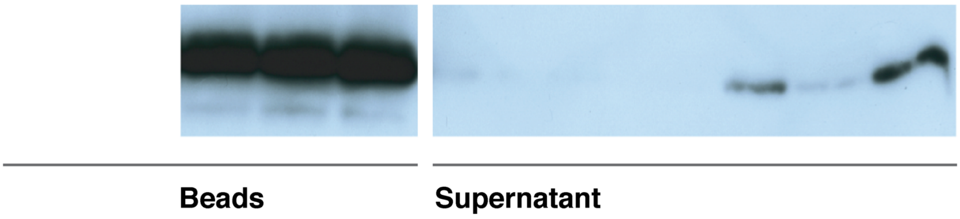

Figure S8: Ensemble experiments of TBP release from the ternary DNA/TBP/Mot1 complex using DNA-coated beads. (**A**) Sequence of the DNA construct used with the attachment site for the bead upstream of the TATA box. (**B**) Immunodetection of TBP on the fraction of beads and in solution upon addition of the components as indicated with the plus signs and in the order shown from top to bottom. The competitor DNA used was reported previously (6).

Suppl. Figure S9

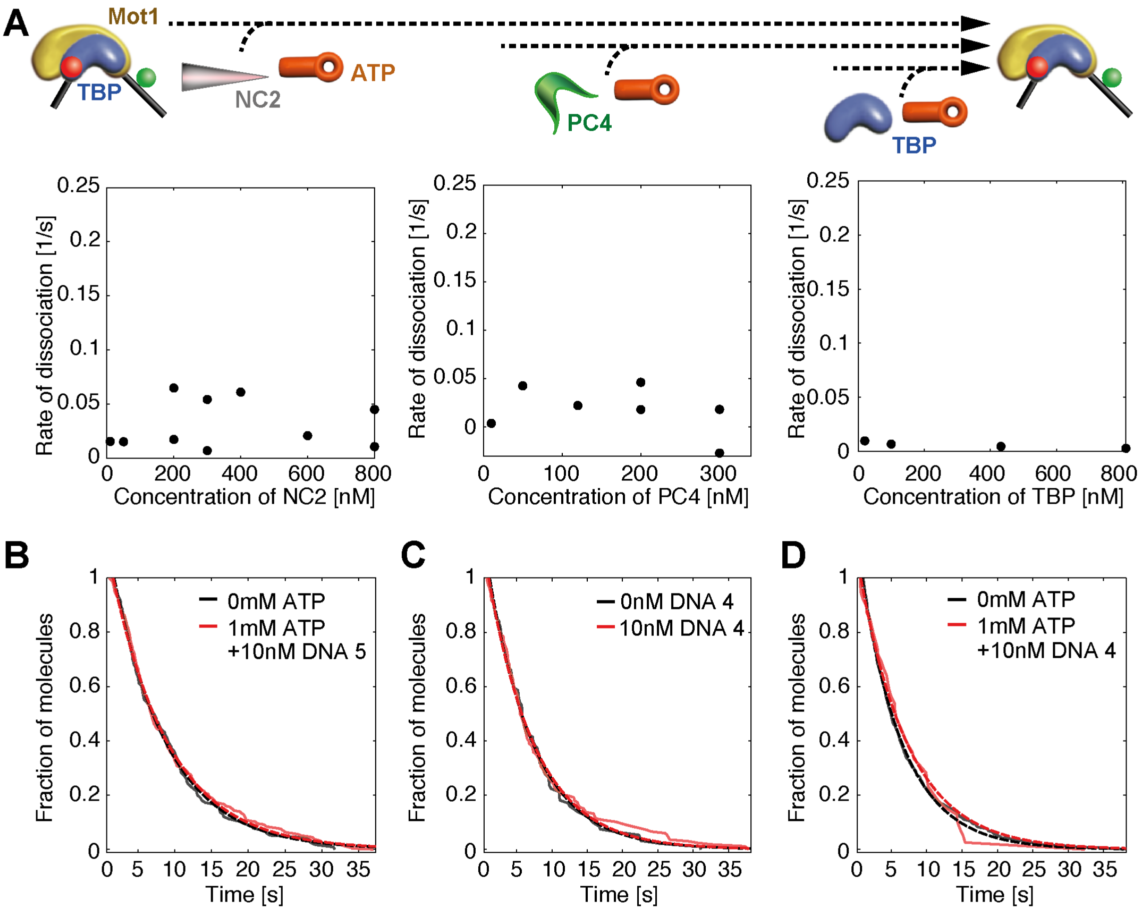

Figure S9. Control experiments for Mot1-induced TBP dissociation from DNA. (**A**) Rate of TBP dissociation from immobilized DNA/TBP/Mot1 complexes in the presence of 1 mM ATP as a function of different concentrations of NC2 (left), PC4 (middle), and TBP (right). (**B-D**) Dwell time histograms of DNA 2/TBP complexes. (**B**) Dissociation of preformed DNA 2/TBP/Mot1 complexes upon combined addition of 1 mM ATP and 10 nM TATA-less DNA (DNA 5). (**C**) Dissociation of preassembled DNA 1/TBP/Mot1 complexes upon addition of 10 nM unlabeled H2B promoter DNA (DNA 4) or a combination of 10 nM unlabeled H2B promoter DNA 4 and 1 mM ATP (**D**).

Suppl. Figure S10

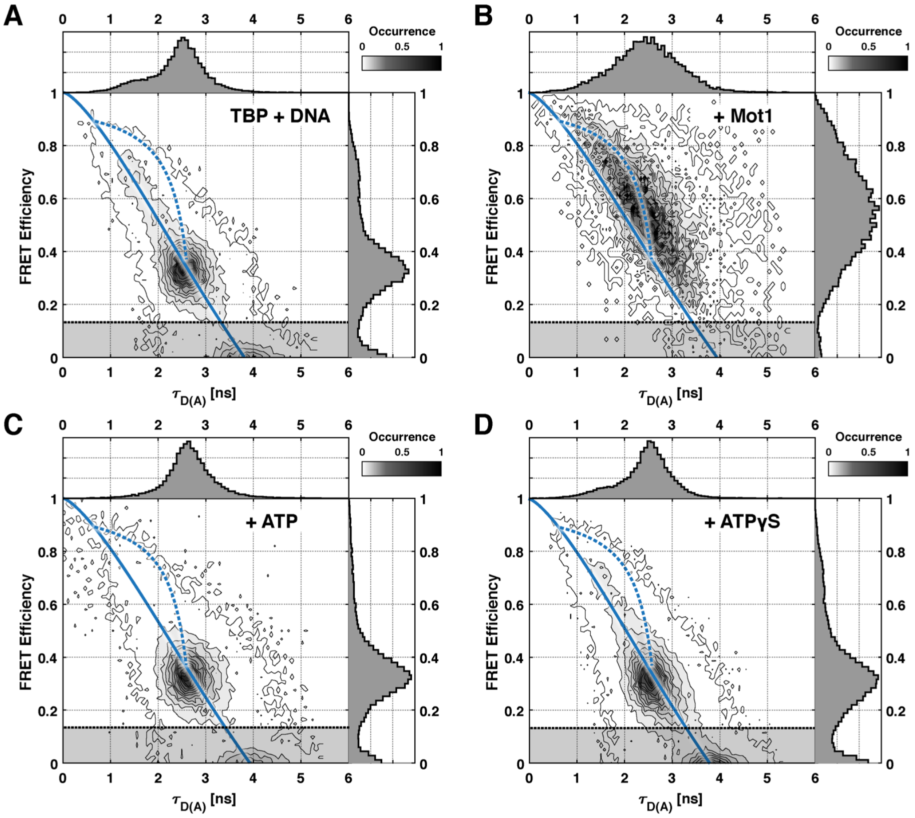

Figure S10. Burst analysis of preincubated DNA/TBP complexes in solution by confocal microscopy. (**A-B**) Two-dimensional histograms of FRET efficiency E versus donor lifetime $\tau_{D[A]}$ for TBP bound to DNA 3 labeled with Atto532 and Atto647N. (**A**) DNA/TBP complex. (**B**) TBP/DNA/Mot1 complex. (**C**) Ternary complex after addition of 1 mM ATP. (**D**) Ternary complex after addition of 1 mM ATPγS. Populations of molecules static in solution during their diffusion time though the focal volume (micro- to milliseconds) are found on the static FRET curve (blue line), a polynomial describing the relationship between the donor lifetime and the FRET efficiency. A deviation to the right of this static FRET line indicates conformational dynamics between different FRET states. Deviations to the left of the line hint at fluorescence quenching.

Suppl. Figure S11

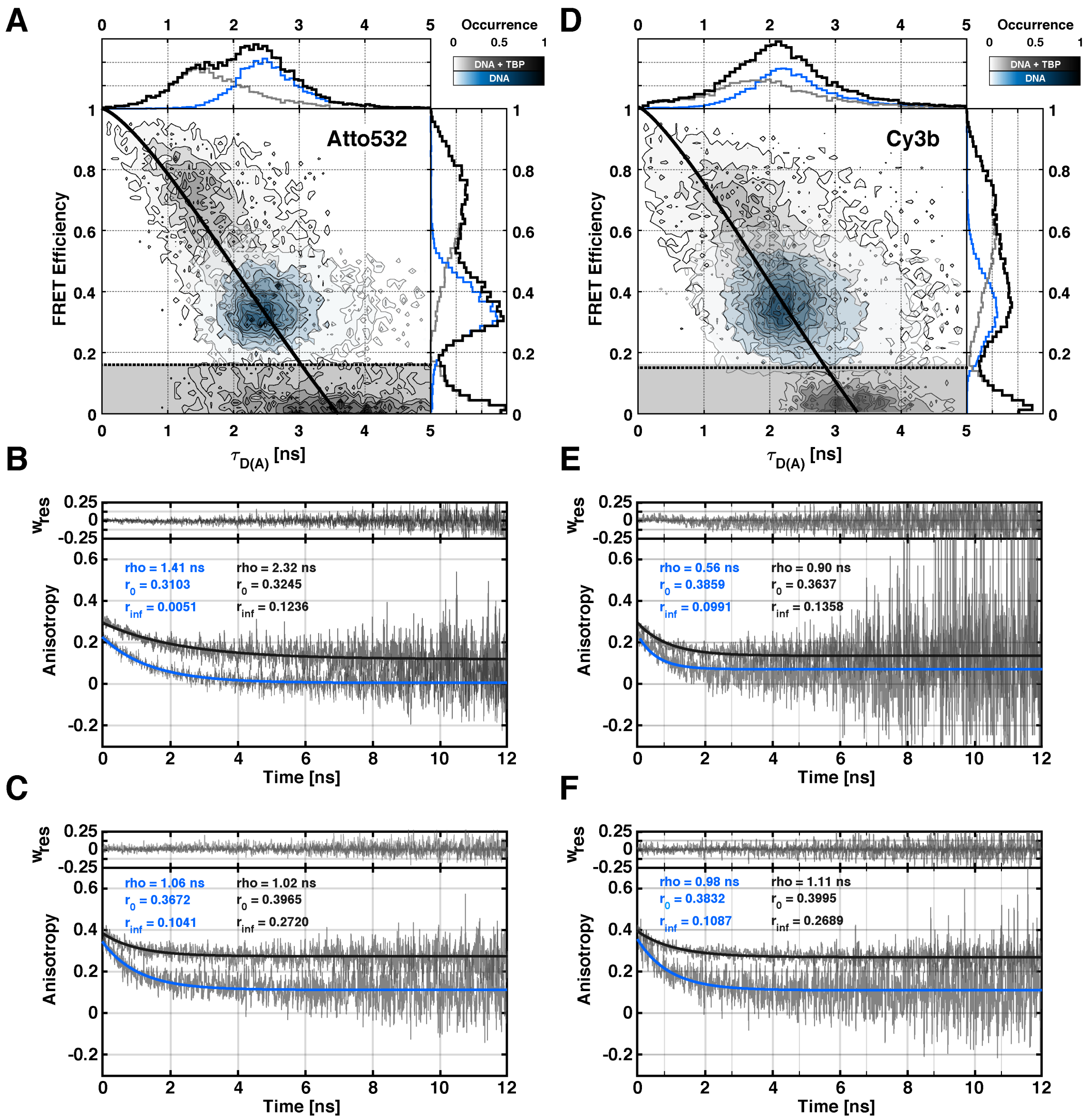

Figure S11. Burst-analysis of dual-labeled H2B promoter DNA 3 for two different fluorophore pairs: (A-C) Atto532/Atto647N and (D-F) Cy3b/Atto647N. DNA 3 was labeled with a donor and acceptor molecule upstream respectively downstream of the TATA box. Binding of TBP at 300 nM leads to a dynamic interconversion of states and restricts the fluorophores mobility. The low FRET state is indicated in blue and the high FRET states are shown in grey. (**A,D**) Life-time vs. FRET efficiency plot (**B,E**) Anisotropy in the absence and presence of TBP for the donor fluorophore Atto532 (**B**) and Cy3b (**E**). (**C,F**) Anisotropy in the absence and presence of TBP for the acceptor fluorophore Atto647N. The time-resolved

anisotropy was analysed using a wobbling-in-cone-approximation, i.e. under the assumption that the fluorophore's rotation is only restricted by the attachment to the DNA. The corresponding fit function is given by $r(t) = (r_0 - r_\infty)\, e^{-t/\rho} + r_\infty$ with r_0 being the intrinsic anisotropy of the molecule, ρ is the rotational correlation time and r_∞ the residual anisotropy due to slow rotational movement of the DNA.

Suppl. Figure S12

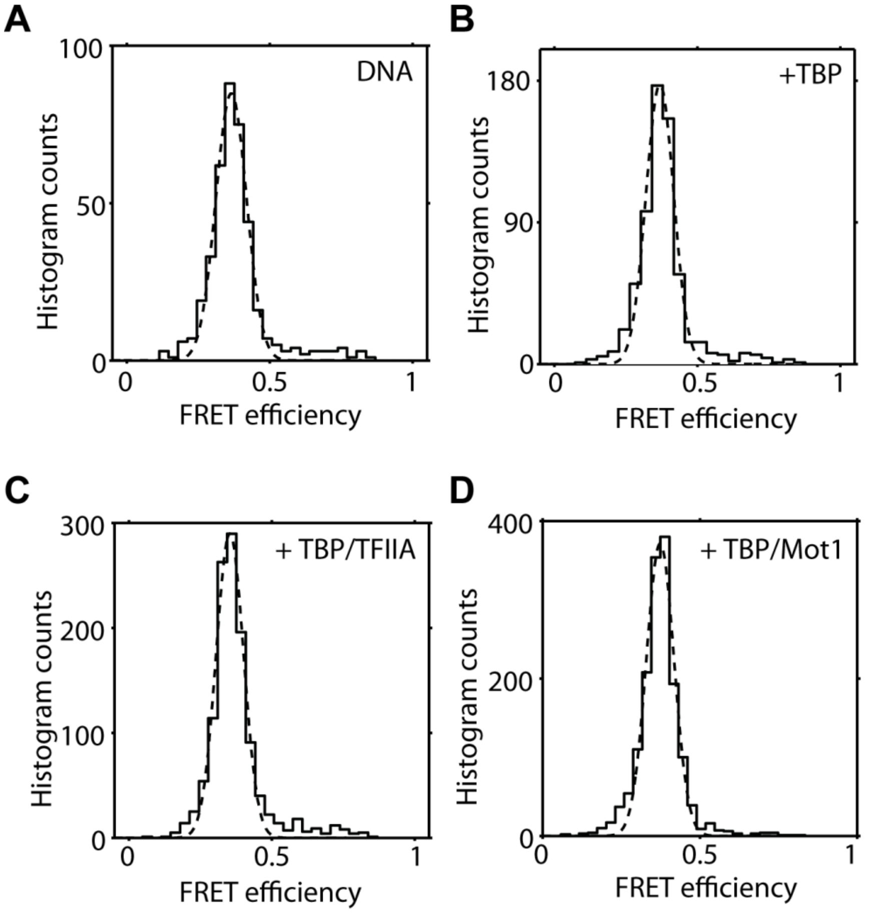

Figure S12. Molecule-wise FRET efficiency histograms of static dual-labeled H2B promoter DNA 3. The promoter DNA 3 was labeled with a donor (Atto532) and acceptor (Atto647N) molecule upstream and downstream of the TATA box. Molecule-wise FRET efficiency histograms of static complexes of DNA alone (**A**) DNA/TBP complexes (**B**), DNA/TBP/TFIIA complexes (**C**), and DNA/TBP/Mot1 complexes (**D**).

Suppl. Table S1

Table S1. Statistics of analyzed molecules in Figures 1- 4 presented in the main text.

Figure 1	C			D		
**DNA**	**+ TBP**	**+ TBP + Mot1**	**+TBP + Mot1 + ATP**	**+ TBP + Mot1**	**+TBP +Mot1 +ATPγS**	**+TBP +Mot1 +ATPγS +ATP**
**Total**	10879	2612	9619	7073	6490	8108
Analyzed	1189	1050	1720	550	550	500
Static	1003	500	1537	500	402	277
Dynamic	157	219	158	135	3	22

Figure 2		A	B	C	D
		**DNA/TBP/Mot1**	**DNA/TBP/Mot1**	**DNA/TBP**	**DNA/TBP/Mot1**
		**+ ATP**	**+ ATP + Mot1**	**+ Mot1 + ATP**	**+ Mot1 + ATPγS**
**Reference**	Total	27995	7817	26908	17604
	Analyzed	12670	7817	3439	6844
	Bleached	3497	1300	3159	674
**Dissociation**	Total	2156	683	2638	1294
	Analyzed	2156	683	2638	1294
	Bleached	1060	212	926	611

Figure 3		A			B	C
		**ATP**	**ADP**	**Mot1**	**+ATP + Mot1 / + ATP**	**+ATPγS + Mot1 / + ATP**
**Reference**	Total	3053 ± 2400	7090 ± 2270	8650 ± 2290	6281	12108
	Analyzed	1515 ± 1100	2510 ± 740	3810 ± 1530	3244	2218
	Bleached	420 ± 299	210 ± 150	820 ± 360	1109	250
**Molecules**	Total	240 ± 150	780 ± 110	860 ± 300	345 ± 10	645 ± 75
	Analyzed	240 ± 150	80 ± 110	860 ± 300	345 ± 10	645 ± 75
	Bleached	120 ± 90	115 ± 90	285 ± 85	236 ± 14	240 ± 50

Figure 4				
**Parameter**	**DNA + TBP**	**+ TF2A**	**+ Mot1**	**+ Mot1 + ATP**
**# of Molecules**				
Total	968	2890	3723	2660
Static	642	1204	2953	2245
Dynamic	286	1582	663	372
Pop. 1	144	964	321	213
Pop. 2	110	223	214	77
Pop. 3	---	131	61	32
**% of Dynamic traces**				
Pop1	14.9%	33.4%	8.6%	8.0%
Pop2	11.4%	7.7%	5.7%	2.9%
Pop3	---	4.5%	1.6%	1.2%
Total	23.1%	45.6%	17.8%	14.0%

Suppl. Table S2

Table S2. Data acquisition settings.

	Figure 1	Figure 2	Figure 3	Figure 4
**Method**	TIRF	TIRF	TIRF	TIRF
**Integration time**	15ms	15 ms	15 ms	15 ms
**Excitation scheme**	G800	R10+G790	R10+G790	G1000
**P(532 nm)**	4mW	8-12mW	8-12mW	20-25mW
**P(647nm)**	---	2-4mW	2-4mW	---
**#Frames**	800	800	800	1000
**#Movies per cond.**	88 ± 12	23 ± 4	25	150
**Repeats**	2-3	2-3	2-3	5-6
	**Figure S2**	**Figure S3**	**Figure S4**	**Figure S5**
**Method**	TIRF	TIRF	PIE-MFD	TIRF
**Integration time**	15ms	15 ms	---	15 ms
**Excitation scheme**	G800	(B1G1)1000	GR	G800
**P(488 nm)**	---	30mW	---	---
**P(532 nm)**	4mW	15mW	100 µW	4 mW
**P(647nm)**	---	---	100 µW	---
**#Frames**	800	2000	3h	800
**#Movies per cond.**	88 ± 12	78 ± 2	---	88 ± 12
**Repeats**	2	---	---	---
	**Figure S7**	**Figure S8**	**Figure S9**	**Figure R1**
**Method**	TIRF	PIE-MFD	TIRF	TIRF
**Integration time**	15 ms	---	15 ms	30 ms
**Excitation scheme**	R10+G790	GR	G1000	R30+G970
**P(532 nm)**	10-12mW	100 µW	20-25mW	25 mW
**P(647nm)**	3-5mW	100 µW	---	10 mW
**#Frames**	800	3h	1000	1000
**#Movies**	25	---	150	80
**Repeats**	6	2	5-6	3

Suppl. Table S3

Table S3. Parameters of the normal-distribution used to fit the molecule-wise FRET histogram shown in Figure 1E and Suppl. Figure S4.

	S_1			S_2			S_3		
	A_1	μ_1	σ_1	A_2	μ_2	σ_2	A_3	μ_3	σ_3
DNA/TBP/Mot1	1	22	4	10	34	13	89	80	4
+ATPγS	42	22	4	50	34	13	8	79	4
+ATP	53	25	4	41	34	13	6	79	6
DNA/TBP/NC2	72	26	5	28	41	13			
DNA/TBP/TFIIA	50	26	4	50	35	12			
DNA/TBP/TFIIA/NC2	71	26	5	29	40	13			

The FRET efficiency is measured between the downstream labeled TATA box and labeled TBP. The mean FRET efficiency μ and standard deviation σ are given in % of the FRET efficiency value.

Suppl. Table S4

Table S4. TBP dissociation from the TATA box – rates of dissociation dependent on concentration.

Figure 2	Initial Complex	Added molecules	Concentration [nM]	Rate of dissociation [1/s]
	DNA/TBP/Mot1	+ ATP / ---		0.0172
	DNA/TBP/Mot1	+ ATP/**Mot1**	3.4	0.0842
	DNA/TBP	+ ATP/**Mot1**	3.4	0.04±0.02
	DNA/TBP/Mot1	+ ATPγS/**Mot1** (seq)	3.0	0.01±0.03
**Figure 3**	**Initial Complex**	**Added molecules**	**Concentration [nM]**	**Rate of dissociation [1/s]**
	DNA/TBP/Mot1	+ ATP/**Mot1**	0	0.017
			1.47	0.029
			2.2	0.060
			3.4	0.034
			3.4	0.059
			3.4	0.084
			5.88	0.119
			7.4	0.093
			10.2	0.203
			11.76	0.115
	DNA/TBP/Mot1	+ ATP/**NC2**	10	0.016
			50	0.015
			50	0.015
			200	0.017
			200	0.065
			300	0.007
			300	0.054
			400	0.061
			600	0.021
			800	0.011
			800	0.045
	DNA/TBP/Mot1	+ ATP/**PC4**	10	0.004
			50	0.043
			120	0.022
			200	0.018
			200	0.046
			300	0.027
			300	0.018
	DNA/TBP/Mot1	+ ATP/**TBP**	20	0.010
			100	0.008
			432	0.005
			810	0.003
	**Initial Complex**	**Added molecules**	**Concentration [mM]**	**Rate of dissociation [1/s]**
	DNA/TBP/Mot1	+ **ADP**	1	0.001
			10	0.013
			100	0.005
	DNA/TBP/Mot1	+ **ATP**	0	0.006
			40	0.024
			100	0.057
			150	0.100
			200	0.148

* ATP/ATPγS was added at a fix concentration of 1mM if not stated otherwise. Varying concentrations of added molecules (bold; column 2) are provided in column 3. Seq: sequentially.

Suppl. Table S5

Table S5. TBP dissociation from the TATA box – rates of dissociation upon subsequent addition of several molecules separately.

Molecule with variable concentration	Concentration	Rate of dissociation [1/s]
ATP (I)	1 mM	0.014
Mot1 (II)	3 nM	0.052
ATP (III)	1 mM	0.017
ATPγS (I)	1 mM	0.050
Mot1 (II)	3 nM	0.026
ATP (III)	1 mM	0.015

Additional references

1. Murakami, K., Tsai, K.-L., Kalisman, N., Bushnell, D.A., Asturias, F.J. and Kornberg, R.D. (2015) Structure of an RNA polymerase II preinitiation complex. *Proceedings of the National Academy of Sciences*, **112**, 13543-13548.

2. Butryn, A., Schuller, J.M., Stoehr, G., Runge-Wollmann, P., Forster, F., Auble, D.T. and Hopfner, K.P. (2015) Structural basis for recognition and remodeling of the TBP:DNA:NC2 complex by Mot1. *eLife*, **4**.

3. Schrödinger, L. (2015), *The PyMOL Molecular Graphics System, Version 1.8*.

4. van Dijk, M. and Bonvin, A.M.J.J. (2009) 3D-DART: a DNA structure modelling server. *Nucleic acids research*, **37**, W235-W239.

5. Kalinin, S., Peulen, T., Sindbert, S., Rothwell, P.J., Berger, S., Restle, T., Goody, R.S., Gohlke, H. and Seidel, C.A.M. (2012) A toolkit and benchmark study for FRET-restrained high-precision structural modeling. *Nat Methods*, **9**, 1218-U1129.

6. Viswanathan, R., True, J.D. and Auble, D.T. (2016) Molecular Mechanism of Mot1, a TATA-binding Protein (TBP)-DNA Dissociating Enzyme. *The Journal of biological chemistry*, **291**, 15714-15726.
